# Water-Induced Transparency Loss in Styrene Butadiene Block Copolymers: Mechanism, Morphology, and Predictive Modeling

**DOI:** 10.1021/acs.macromol.5c01354

**Published:** 2025-07-30

**Authors:** Jenoff E. De Vrieze, Michiel Verswyvel, Kinza Y. Ghulam, Bart-Jan Niebuur, Tobias Kraus, Markus Gallei, Norbert Niessner

**Affiliations:** † Global R&D Laboratories, 434867 INEOS Styrolution Group GmbH, Mainzer Landstraße 50, 60325 Frankfurt, Germany; ‡ Polymer Chemistry, 9379 Saarland University, Campus C4 2, 66123 Saarbrücken, Germany; § INM—Leibniz Institute for New Materials, Campus D2 2, 66123 Saarbrücken, Germany; ∥ Colloid and Interface Chemistry, Saarland University, Campus D2 2, 66123 Saarbrücken, Germany; ⊥ Saarene, Saarland Center for Energy Materials and Sustainability, Campus C4 2, 66123 Saarbrücken, Germany

## Abstract

Water-induced transparency loss in styrene–butadiene block copolymers (SBCs) has been investigated under a variety of conditions. Consistent with earlier work on homopolymers, the opacity after prolonged water exposure is expected to be caused by water clustering, which results from stronger water–water than water–polymer interactions. The water clusters distort the surrounding polymer matrix, causing local changes in the refractive index. It was found that the hard phase has only a minor contribution to the transparency loss, while the rubbery phase appears to be the major contributor. However, the loss of transparency was found not to be directly proportional to the volume of the soft phase, and a significant effect of the block copolymer morphology was observed, which was confirmed by a series of transmission electron microscopy and SAXS measurements. This effect is particularly evident in the transition from a continuous hard phase through a co-continuous morphology to a continuous soft phase. The acquired insights were subsequently used to predict long-term optical performance in SBCs to provide a tool in product development. Loss of transparency predictions was proven to be adequate through a classical regression-extrapolation approach using a limited data set, accurately simulating performance beyond 2600 h exposure time using only 600 h of measurement time. Additionally, it was shown that artificial neural networks could provide a solid tool in predicting performance even prior to synthesis, granted that the selection of descriptors is complete and the appropriate amount of data is supplied with a proper spread over the descriptor space.

## Graphical abstract

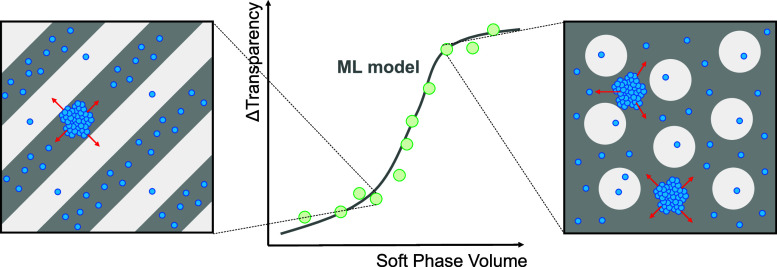

## 1 Introduction

Understanding the effect of moisture on the polymer performance is essential when designing materials for medical devices and tools,^1^ food packaging,^2^ electronics,^3^ and many other applications. The diffusion of water into polymeric materials can strongly influence both the optical and mechanical properties. In polystyrene (PS), for example, a slightly opaque, mist-like pattern is observed when the material is soaked with water at elevated temperatures for more than 10 h.^4^ In addition, water absorption has a significant effect on its mechanical properties, reducing both cracking and yield stress.^5^ More severe loss of optical performance was observed for thin films of styrene-acrylate copolymers,^6^ where some copolymers even completely lose their transparency after 12 h in 70 °C water.^7^

As a result, the diffusion of water in polymeric materials and its effect on both mechanical and optical properties have been the focus of numerous research activities over the past decades.^8,9^ The diffusion and absorption rate of water in polymers was measured for a wide selection of materials and was concluded to be rather anomalous.^10–12^ Usually, the diffusion coefficients of organic vapors increase as the penetrant concentration is raised. However, in the case of water diffusion, it is observed that at high activity, the diffusion coefficient decreases with concentration. It was proposed that these observations are the results of the so-called clustering of water molecules. At low concentrations, water molecules show a classic Fickian diffusion through polymeric materials. When the concentration increases, however, water molecules will start to interact with each other through hydrogen bonding and arrange in clusters. This is the result of a relatively strong water–water interaction compared to generally poor water–polymer interaction. As a consequence, the amount of water available for diffusion is lower than its actual concentration, lowering the apparent diffusion coefficient.^13^ Based on these observations, the Zimm−Lundberg clustering function was developed in an attempt to quantify the size of water clusters via estimation of the probability of finding nearby water molecules.^14,15^ In a study by Davis and Elabd, the existence of such clusters in PS and poly­(methyl methacrylate) was proven using IR techniques.^16^ However, it was found that the aforementioned Zimm−Lundberg clustering function generally underestimates cluster formation as the material is generally in a nonequilibrium state due to stress-induced relaxation.^17^

Given the abovementioned proposed mechanism behind these observations, a strong difference in behavior between glassy and rubbery polymers can be expected. Polymers below their glass transition temperatures are stiff and have low segmental mobility. This means that clusters will form almost exclusively in existing voids and defects in the material. The size of the clusters is, therefore, limited by the size of these microcavities. For polymers above their glass transition temperature, however, the picture is drastically different. These materials are generally less dense and have a high segmental mobility. As a result, clusters can initiate anywhere in the material and their growth is less constrained by the polymer matrix.^18^ This immediately explains the big contrast between, for example, PS and polyisoprene. While PS only shows a slightly hazy mist-like change in transparency when exposed in 60 °C water,^4,19^ polyisoprene becomes completely turbid.^20^ Significant differences also exist between polar and nonpolar polymers. It was shown that water clusters are most likely to occur near polar groups and that hydrogen bonding interactions have a significant effect on diffusion rates and cluster size.^21,22,26^ In addition, different states of water molecules are observed due to different levels of interaction with the polar sites.^24^

Even though the mechanism of water absorption in homogeneous polymeric materials is well-understood, there is a lack of knowledge about how water absorption and diffusion occurs in multiphase polymers and how it is affected by the morphology of the nanophase-separated structures. Komarova et al. observed complete turbidity of styrene–butadiene block copolymers (SBCs) after soaking samples in water at 37 °C for 60 days and related this to the presence of residual lithium salts and antioxidants.^25^ Jacobs and Jones studied water absorption in styrene–(ethylene/butylene)–styrene block copolymers and concluded that there are two regions of absorption.^23^ In the first region, moisture is absorbed in both the dense hard phase and less dense soft phase. When the soft phase is saturated, absorption continues in the hard phase.^25^ Pissis and co-workers, on the other hand, concluded that absorption almost exclusively occurs in the soft phase and that, similar to homogeneous polymeric materials, clustering occurs from a certain critical water activity onward.^27^ Oparaji and co-workers studied the effect of morphology and crystallization in poly­(styrene-*b*-ethylene oxide) block copolymers on their water absorption behavior.^28,29^ They showed that morphology does have a clear effect and that for a morphology with poly­(ethylene oxide) cylinders dispersed in a PS matrix, the diffusion is very similar to homopolymer PS. In a lamellar morphology, on the other hand, the diffusion is more similar to diffusion in pure poly­(ethylene oxide). Recently, Plank et al. investigated the influence of 3 vol % of water in BCP casting solutions based on PS-*b*-PBd switching from well-defined hexagonal cylinders or lamellae as the equilibrium morphologies to a homogeneous, nonequilibrium hexagonally perforated layer morphology and a gyroid structure by introducing water.^30^

This work aims to further elucidate the effect of morphology on the absorption and diffusion of water in two-phase BCPs as well as the influence of various environmental factors. BCPs with special molecular architectures, such as branching points or tapered segment,^31–35^ exhibit fascinating morphologies. The presence of water in such BCPs is of special interest, as many polymers in our daily life, especially those used in medical care, are sterilized with steam or salty water solutions. In other applications, thermoplastic elastomers or mixtures thereof are subjected to dishwashing. As a result, the absorption of water can lead to a loss of optical transparency, and it is essential to understand the general mechanisms that occur during prolonged immersion in water at elevated temperatures. The observations are subsequently rationalized by transmission electron microscopy (TEM) and small-angle X-ray scattering (SAXS) measurements on a set of linear BCPs. The final part of this paper is dedicated to predictive modeling by using the knowledge gained from the experimental study to provide a framework for future product development. Throughout the work, the focus is exclusively on SBCs derived by carbanionic polymerization as they combine a high level of polymerization control with a highly customizable morphology.^36^ In general, star-shaped and branched polymers were selected as they provide an ideal balance between processability (short chains = low viscosity) and mechanical strength (long chains = high entanglements). These star-shaped polymers are representative of commercial products used in a variety of applications from shrink sleeves to high impact injection molding grades.^37,38^ In addition, the use of SBCs in moist and wet environments is of particular interest due to their widespread use in medical, food packaging, and electronic applications.

## 2 Experimental Section

### 2.1 Chemicals

The styrene and butadiene monomers (unstabilized), cyclohexane, isopropanol, epoxidized soybean oil (ESBO), and potassium *tert*-amylate (5 wt % in cyclohexane) were acquired directly from the commercial INEOS Styrolution Styrolux production plant. *sec*-Butyllithium was purchased as a 12 wt % solution (in cyclohexane) from Livent. 1,1-Diphenylethylene (DPE), Irganox 1010, and Irgafos 168 were purchased from BASF, and Sumilizer GS was acquired from Sumitomo Chemical. Toluene (>99.7%), isoprene (>99.8%), and tetrahydrofuran (THF) (>99.7%) were purchased at VWR.

### 2.2 Polymerization

To investigate the role of polymer architecture and morphology on water absorption and clustering, different linear and star-shaped polymers were synthesized. An overview of the block structures and recipe details is provided in Tables 1 and 2, respectively. Further information on the characterization and an example of the general BCP synthesis and coupling strategy are depicted in Table S1 and Scheme S1 in the Supporting Information (SI) based on ST-3.

**Table 1:**
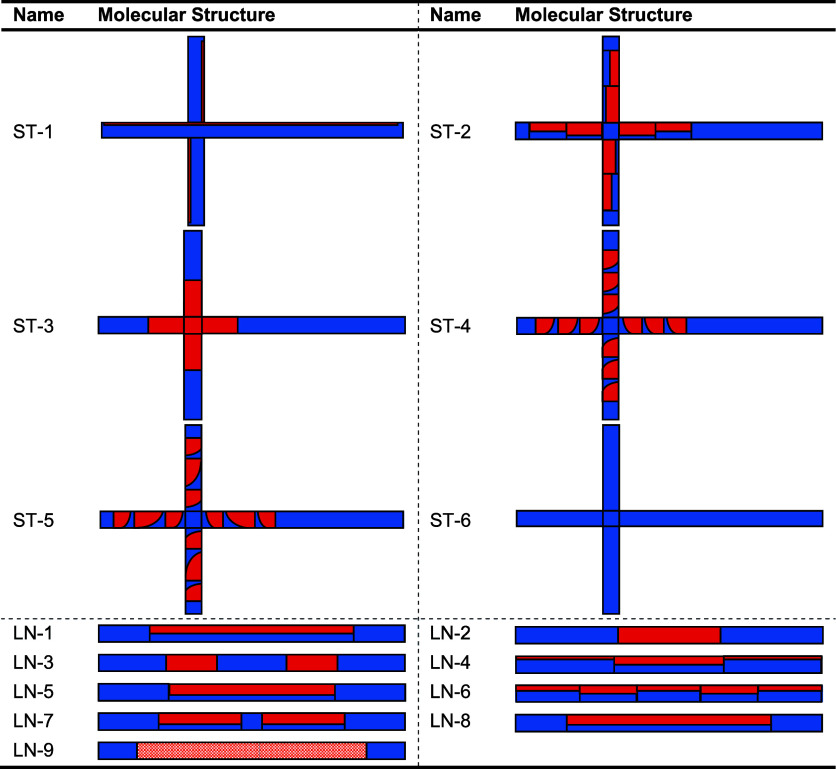
Molecular Structure and Description of the Different Synthesized Samples^a^

a Red represents butadiene, textured red represents isoprene, and blue represents styrene. Details on the composition and the ratio of short-to-long arms are outlined in Table 2.

**Table 2:** Overview of the Polymerization Recipes Used During This Study^c^

		initiations			block 1		block 2		block 3		block 4		block 5	
name	coupled	*N*	Pos	*R*_ini_	S (%)	B (%)	S (%)	B (%)	S (%)	B (%)	S (%)	B (%)	S (%)	B (%)
ST-1	Y	2	3	0.8	5	0	61.5	8.5	2^b^	0	11.5	1.5		
ST-2	Y	2	2	2.5	34	0	21	0	7	7	9	19	3	0
ST-3	Y	2	2	4.4	48	0	27	0	0	25				
ST-4^a^	Y	2	2	2.0	20	0	30	0	4	12	4	13	4	13
ST-5^a^	Y	2	2	1.09	15	0	17	0	11	15	15	10	4	13
ST-6	Y	1			100	0								
LN-1	N	1			16	0	34	34	16	0				
LN-2	N	1			33	0	0	34	33	0				
LN-3	N	1			22	0	0	17	22	0	0	17	22	0
LN-4	N	1			24	8	18	18	24	8				
LN-5	N	1			23	0	20	34	23	0				
LN-6	N	1			16	5.3	9	9	16	5.4	9	9	16	5.3
LN-7	N	1			16	0	13	17	8	0	13	17	16	0
LN-8	N	1			16	0	33	35	16	0				
LN-9	N	1			12.5	0	0	75	12.5	0				

a In contrast to the other polymers synthesized in this study, these polymers were produced with tapered (S/B) blocks instead of random (S/B) blocks.

b The 2 wt % PS block (*Đ* ≈ 1.09) was included to ensure efficient initiation of the subsequent long chain SBC (*Đ* ≈ 1.10), confirming that the initiation and transfer to the mixed block were successful.

c Coupled products were terminated using ESBO, uncoupled products were terminated with isopropanol. In case the recipe comprises multiple initiations, the position number of, e.g., the second initiation indicates the block following the second initiation. The initiation ratio represents the molar ratio of the second initiation with respect to the first initiation.

All polymerizations were performed in a 50 L pilot reactor using the same general recipe; the structural details of each of the recipes are outlined in Table 2. First, the reactor was loaded with about 15 kg of cyclohexane and heated to 50 °C after which 2 mL of DPE was added and the solution was titrated with *sec*-butyllithium until color change. After that step, the desired amount of butyllithium solution was dosed in the first initiation step, followed by a sequence of different monomer additions and, in the case of multiple initiations, *sec*-butyllithium additions. Monomers were dosed in such a way that the total polymer weight amounted to 5 kg. The total amount of butyllithium was derived from a predefined molecular weight to obtain a target melt flow index. In the case of linear polymers (labeled LN in Table 2), polymerization was terminated with isopropanol. When star polymers were targeted (labeled ST in Table 2), the living chains were coupled with ESBO using a 12.07 mmol_*sec*‑BuLi_: ml_ESBO_ ratio. The styrene–butadiene mixed blocks were randomized by dosing potassium *tert*-amylate (KtA) prior to each initiator dosing (except for titration), a molar Li/K ratio of 35 was always targeted. In recipes ST-4 and ST-5, the addition of KtA was omitted, and hence tapered block structures were synthesized.

After polymerization and termination or coupling, the polymer solution was transferred to a stabilization vessel in which the lithium salts were neutralized by dosing 10 g of H_2_O and 8.3 g of CO_2_, resulting in the formation of lithium carbonate and/or lithium hydrogen carbonate. Subsequently, an additive package consisting of 0.2 wt % Irganox 1010, 0.15 wt % Irgaphos 168, and 0.15 wt % Sumilizer GS with respect to the total mass of polymer was added. The polymer solution was subsequently extruded at 180 °C with a 6 kg/h feed rate and granulated into cylindrical pellets. The success of the polymerization was verified through gel permeation chromatography (GPC) and melt flow index measurement. An overview is provided in Table S1. The granules were subsequently injection molded into 2 mm transparent plaques using an Arburg 320S machine with a melt temperature of 180 °C and a mold temperature of 50 °C. After injection molding, the plaques were allowed to relax during at least 24 h at room temperature.

### 2.3 Instrumentation

The molecular weight of the resulting polymers was determined by GPC using an Agilent 1260 infinity SEC setup. The samples were dissolved in THF with a final concentration of 1 mg/mL and dried toluene as marker. Measurements were performed with 4 columns and using 2 detectors. The first column is a Waters APC guard column of 900 Å, the second column is a Waters APC XT column of 900 Å, the third column is a Waters APC XT column of 450 Å, and the fourth and last column is a Waters APC XT column of 125 Å. The 2 detectors are an Agilent 1260 infinity refractive index detector and Agilent 1200 Infinity series variable wavelength detector. THF was the eluent at a 0.5 mL/min flow rate. All measurements relate to a PS calibration kit purchased from PSS. The data analysis was performed with WinGPC (PSS).

SAXS measurements were performed on a Xeuss 2.0 instrument (Xenocs SAS, Grenoble, France). A collimated beam from the K_α_-line of a copper X-ray source with a wavelength of λ = 1.54 Å was focused on the sample with a spot size of 0.25 mm^2^. Two-dimensional scattering images were recorded using a Pilatus 300 K detector with pixel sizes of 0.172 mm × 0.172 mm and a sample-to-detector distance of 2496 mm, calibrated using a silver behenate standard. The dry samples with thicknesses of ∼1 mm were placed directly in the beam, i.e., without using a sample container, and were measured under vacuum conditions at around 27 °C with an acquisition time of 3600 s. In all cases, the scattering images showed no sign of anisotropic scattering. Therefore, they were azimuthally averaged to obtain *I*(*q*). Here, *q* is defined as *q* = 4π × sin­(θ/2)/λ with θ being the scattering angle. Sample LN-7 was measured under hydrated conditions, too. For this, the sample was placed at the bottom of a borosilicate capillary with an inner diameter of 1.5 mm, while the top part of the capillary was filled with water, kept at a position by capillary forces, to expose the sample to a high relative humidity. The capillary was sealed at the top using epoxy resin adhesive. After ∼15 h of equilibration time, a measurement at the sample position with an acquisition time of 3600 s was performed. In the second experiment, the same sample was immersed completely in water inside of a sealed borosilicate capillary. After ∼15 h of equilibration time, the second measurement with an acquisition time of 3600 s was performed.

The melt volume rate was measured (in cm^3^/10 min) at 200 °C and with a 5 kg weight using a Zwick Aflow extrusion plastometer according to DIN ISO 1133.

Modulated differential scanning calorimetry (MDSC) measurements were performed using the TA Instruments Q20 device. 15 mg of granulate was loaded into a pan and heated from −85 to +200 °C using a heat rate of 2 °C/min with a modulation of ±1.5 °C every 60 s.

Dynamical mechanical analysis (DMA) was measured using a TA Instruments Q800 device using a single cantilever clamp. A rectangular specimen is used (17.1 mm × 10.07 mm × 4.23 mm) that is produced by cutting a Charpy bar (ISO 179-1) in half. The sample is first equilibrated at −120 °C for 1 min and subsequently heated to 120 °C with a ramp of 2 °C/min, where it remained for 1 min. The measurement is performed with an amplitude of 25 μm and a frequency of 1 Hz.

The TEM analyses were performed with an FEI Tecnai Spirit Twin equipped with an Olympus-SIS MegaView G2 CCD camera. The images were recorded with binning 1 so that the images contain 1374 × 1030 pixels (without marker). The TEM samples were prepared by making a microtome cut from a 2 mm thick optical plaque. The polybutadiene fraction of the block copolymers was stained by placing a droplet of OsO_4_ on the microtome cut samples. Additional TEM images were obtained using a JEOL JEM-2100 LaB_6_ electron microscope (JEOL, Tokyo, Japan) with 200 kV acceleration voltage or a JEOL JEM-F200; 0.14 nm line resolution and a Gatan Orius SC1000 camera (Gatan, Pleasanton, CA, USA) in the brightfield mode. The contrast and smoothness of the images were adjusted with ImageJ 1.53 k (NIH, USA). Domain sizes were also measured with ImageJ as a mean value of 20 points. Here, ultrathin sections (40 nm) were prepared with an ultramicrotome (Reichert Ultracut by Leica Microsystems, Wetzlar, Germany) and placed on a copper grid. The thin films were put on a TEM Grid and stained with OsO_4_ for 5 min. Afterward, the films were coated with carbon.

To quantify the lithium recovery efficiency of the washed polymer solution, ICP-OES analysis was performed on the water sample using a PerkinElmer Optima 8300. The measured lithium concentration and removed water volume were then compared with the initial lithium dosage in the reactor to calculate the removal rate.

### 2.4 Experimental Setup

A picture of the experimental setup is provided in Figure S1. The 2 mm optical plaques produced via injection molding were submerged in a 5 L beaker and filled with water. To prevent the plaques from reaching the surface, they were placed in smaller beakers, which were positioned on the bottom of the large beaker. The water surface was kept sufficiently low to prevent the plaques from leaving the small beakers and from floating. The water in the beaker was stirred at 690 rpm and heated via a heating plate. Under some conditions, salt (NaCl) was added to the solution, which was done prior to submerging the plates. At predetermined timestamps, the plaques were removed from the solution and externally dried in order to measure the loss of transparency and relative mass increase. The plaques were subsequently resubmerged in the solution. Loss of transparency as a function of time was calculated using eq 1:

1 ΔTrans(t)=Trans(t=0)−Trans(t)

in which transparency (Trans) was measured using the haze guard plus device (BYK instruments) with a white ceramic background.

The relative mass increase due to water absorption was calculated using eq 2:

2 $$\Delta m=\frac{m\left(t\right)-m\left(t=0\right)}{m\left(t=0\right)}$$

in which the mass of the plaque (*m*) was measured using an Ohaus Explorer EX324M/AD with an accuracy of 0.1 mg.

### 2.5 Modeling Details

#### 2.5.1 Regression Extrapolation

A regression-extrapolation approach for predictive modeling was applied by using an in-house developed python code. In the code, three different functions, describing a nonlinear decay, were evaluated (Table 3). The code was constructed by gradually adding measurement points to the model. After each measurement point addition, a new regression was performed for each of the functions in Table 3. Regression to the functions was performed using the curve fit function in SciPy.

**Table 3:** Functional Descriptions Used to Perform a Regression-Based Extrapolation in Predictive Modeling of Loss of Transparency in SBC Optical Plaques

functional description	equation
rational decay	3 $\Delta Trans=a-\frac{bt+c}{dt^{2}+et+f}$
power law	4 $\Delta Trans=at^{b}$
exponential decay	5 ΔTrans=a−b·exp(−ct)

The performance of the different functions was evaluated by using two different criteria. For each point added to the data set, the absolute prediction error at 2616 h was measured. This absolute prediction error was defined as the absolute difference between the predicted and measured transparency at 2616 h. This provides a tool to determine how much measurement time would be required to accurately predict the optical performance of the material after just ∼3 months of immersion under the conditions studied. For each point added to the data set, the statistical performance of the different models was compared using an *F*-test. This test was used to assess whether the model with a greater number of parameters provided a significantly better fit.

#### 2.5.2 Artificial Neural Network

An artificial network was constructed to estimate the time-dependent transparency loss as a function of the soft phase volume. The artificial neural network was constructed using the MLPRegressor functionality of sklearn in python and was trained using the time-dependent transparency data at 55 °C measured for structures LN-1 to LN-7 measured over a period of 2616 h. An adequate network architecture was determined by evaluating different neuron activation functions in combination with different numbers of hidden layers and different numbers of neurons per layer. The number of hidden layers was varied between 1 and 3, the number of neurons was varied between 1 and 4 (not higher than twice the number of input parameters). In both evaluation and simulation, the limited-memory BFGS (lbfgs) solver was used with maximum 10,000 iterations. The performance of the candidate models was evaluated by calculating the mean and median scores of 100 repeated evaluations as well as evaluating the predictability. For each repeated evaluation, the data set was shuffled and 20% of the data was removed for testing. The model was subsequently trained with the remaining 80% of data, and the score was determined using the 20% removed data. An overview of the results of this model analysis is shown in Figure S2. The best overall performance (score and predictability) was observed for a network based on the ReLU activation function that uses two hidden layers with 4 neurons in the first layer and 3 neurons in the second. The L_2_ regularization parameter (α) was kept at its default value of 0.0001 throughout.

## 3 Results and Discussion

### 3.1 Effect of Lithium Salts and Stabilizers

In past work on the effect of water on the optical and mechanical performance of SBCs, it was assumed that the presence of lithium salts and/or phenolic stabilizers is mainly responsible for the absorption and clustering of water.^21^ To assess any effects related to the presence of Li ions, recipe LN-1 was repeated two times. In this first iteration, the exact sequence as presented in section 2.2 was performed. A schematic overview of the polymer synthesis is provided in Scheme S1. In the second recipe, sequence 2.2 was followed by a water-washing step to remove lithium ions from the polymer. The details of this washing step and results of the ICP measurement are outlined in SI section 4. The ICP measurement confirmed that approximately 95% of the lithium ions were removed from the polymer product. Both samples were subsequently injection molded and submerged in 25 and 55 °C water for 336 h together with a sample of anionically synthesized PS (ST-6). The loss of transparency of each sample was measured at specific timestamps, the results of which are presented in Figure 1.

**Figure 1:**
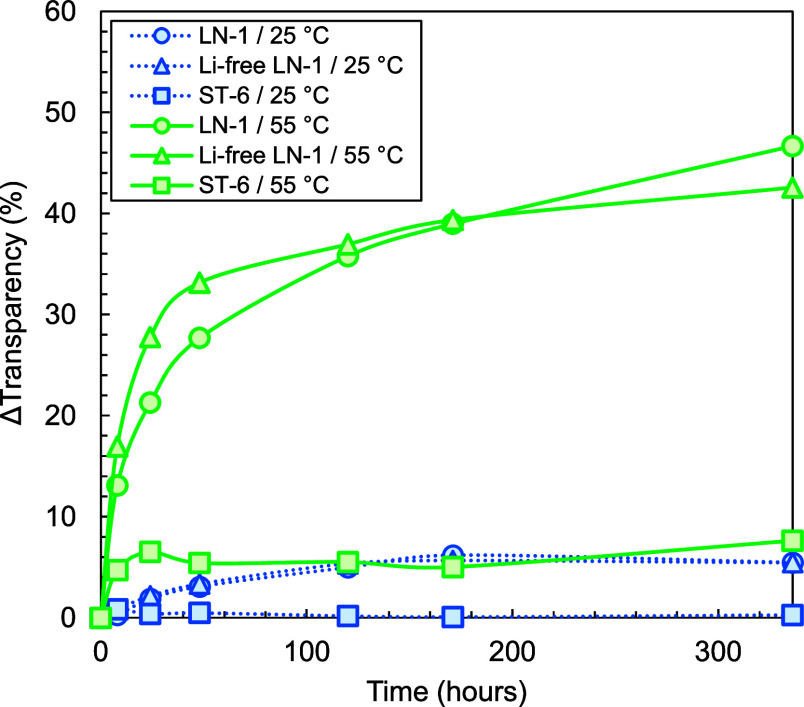
Loss of transparency at 25 °C (dotted blue line) and 55 °C (solid green line) in water as a function time for linear SBC recipe LN-1 both with the original Li content (▲) and with a 95% reduced Li content (●) as well as anionically synthesized PS (■).

At 25 °C, LN-1 shows a small initial increase, followed by a plateau of transparency loss. There is no notable difference between the standard and the “Li-free” sample. At 55 °C, the standard and Li-free samples follow the same trend, and the Li-free sample even shows a slightly higher loss of transparency at 336 h. This, however, might be related to experimental variation or initial water content. PS synthesized via anionic polymerization, on the other hand, shows no measurable transparency loss at 25 °C and only a slight loss of transparency at 55 °C that remains constant after a few hours. Similar to previous reports,^16^ a mist-like pattern was observed for the PS sample. Interestingly, the samples of LN-1 become uniformly opaque, while the ST-6 samples (anionically polymerized PS) show the aforementioned mist-like pattern that concentrates around existing defects originating from injection molding (see Figures S3–S5). These observations support the abovementioned hypothesis, which states that water can form clusters anywhere in polymers or polymer phases above their glass transition temperature,^15,38^ while below the glass transition temperature, they will only form in preexisting cavities and defects.

To investigate the potential effect of antioxidants, 2 mm plaques of a stabilizer-free LN-1 product were produced and subjected to the same conditions as those for the plaques above. However, direct measurement of transparency was difficult as the plaques showed a very heterogeneous transparency due to cross-linking during extrusion and injection molding. A qualitative investigation of the loss of transparency was therefore carried out, and the optical appearance was compared with the standard LN-1 sample (see Figure S6). It is clear that the additive-free sample loses transparency in a similar time frame as the standard sample, indicating that the additive is clearly neither the root cause nor does it have a major effect on the optical performance.

### 3.2 Effect of the Polymer Recipe

To study the effect of the polymer recipe, optical plaques of 7 different recipes (ST-1 to LN-1 in Table 2) were produced and submerged for 360 h in water under different conditions: 25 °C, 55 °C, and 55 °C with 1 wt % of NaCl, which is close to the isotonic concentration of NaCl (0.9 wt %) for medical applications. The latter is of particular interest for a large number of medical applications. The relative mass increase and loss of transparency were measured for each of the samples at predetermined timestamps. Figure 2 comprises the consolidated data from this experiment and shows how the loss of transparency relates to water absorption. At room temperature, absorption is limited, and all samples retain most of their transparency during prolonged water exposure. When the temperature is increased to 55 °C, however, absorption is more substantial and the transparency drops significantly. Figure 2 indicates that independently of the exposure conditions, transparency loss strongly increases with water absorption, which supports the aforementioned cluster theory. It is also noteworthy that the addition of NaCl seems to somewhat hamper absorption which is expected to be the result of an osmotic effect. Moreover, the addition of NaCl seems to, in some cases, even lower the transparency loss for the same degree of water absorption, indicating an additional effect.

**Figure 2:**
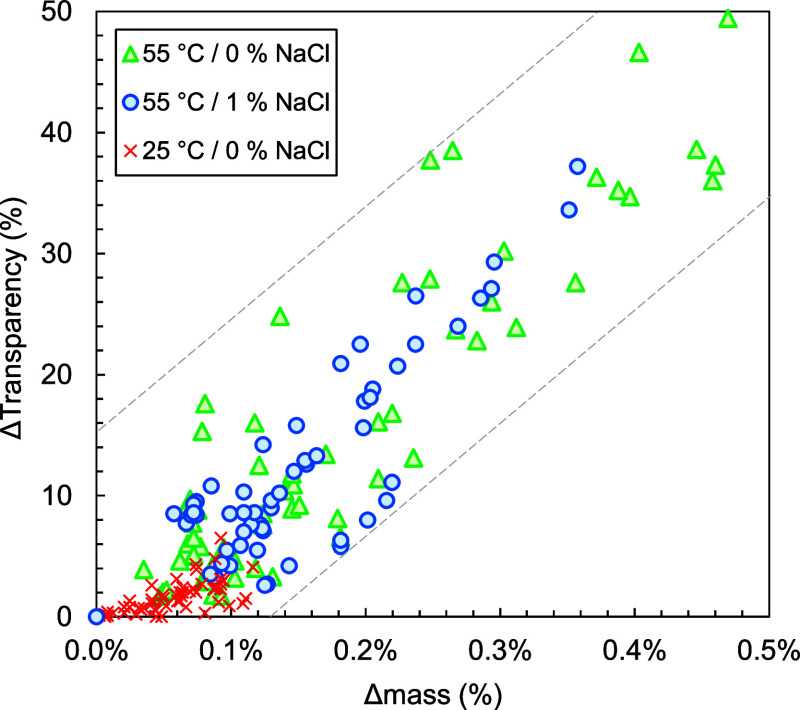
Loss of transparency as a function of relative mass increase for 2 mm optical plaques of the samples according to recipe ST-1, ST-2, ST-3, ST-4, ST-5, ST-6, and LN-1 measured at 25 and 55 °C in demineralized water and measured at 55 °C in demineralized water with 1 wt % of NaCl. Measurements were taken after 4, 8, 24, 72, 97, 192, 240, and 360 h.

The different polymer structures show a clear difference in the relationship between transparency loss and water absorption (Figure 3A). For each sample, an S-shaped curve can be identified. This is not surprising, as at low water absorption, the penetrant concentration will be too low to form sizable clusters. However, both the slope and the inflection point of these S-curves seem to heavily depend on the recipe. To rationalize this change in slope, the theoretical soft phase volume of each of the samples was calculated using the Fox equation (see Table S2). The calculated values are indicative of a general trend in which the degree of water absorption increases with an increasing soft phase volume. This is not surprising as diffusion and absorption in a less dense soft phase are significantly faster than that in a dense rigid polymer or phase. The soft phase volume is not the only factor, however, as polymer LN-1 shows a drastically higher water absorption and transparency loss for the same exposure time as ST-5, even though they both have an identical theoretical soft phase volume (68 wt %). In addition, polymer ST-2 shows a slightly higher water absorption and transparency loss as ST-5, even though it has a significantly lower soft phase volume (45 wt %). Both observations are indicative of a morphological effect. One could argue that this is the result of a lower effective soft phase volume in the case of polymer ST-5 due to mixing of the styrene-rich part of the tapered block into the hard phase. However, DMA showed that the measured soft and hard phase glass transition temperatures for ST-5 have near-perfect correspondence to the theoretical ones: −54 °C/97 °C and −57 °C/100 °C, respectively (see Figure S8). This indicates that mixing is limited and that the theoretical and actual soft phase volumes show little difference.

**Figure 3:**
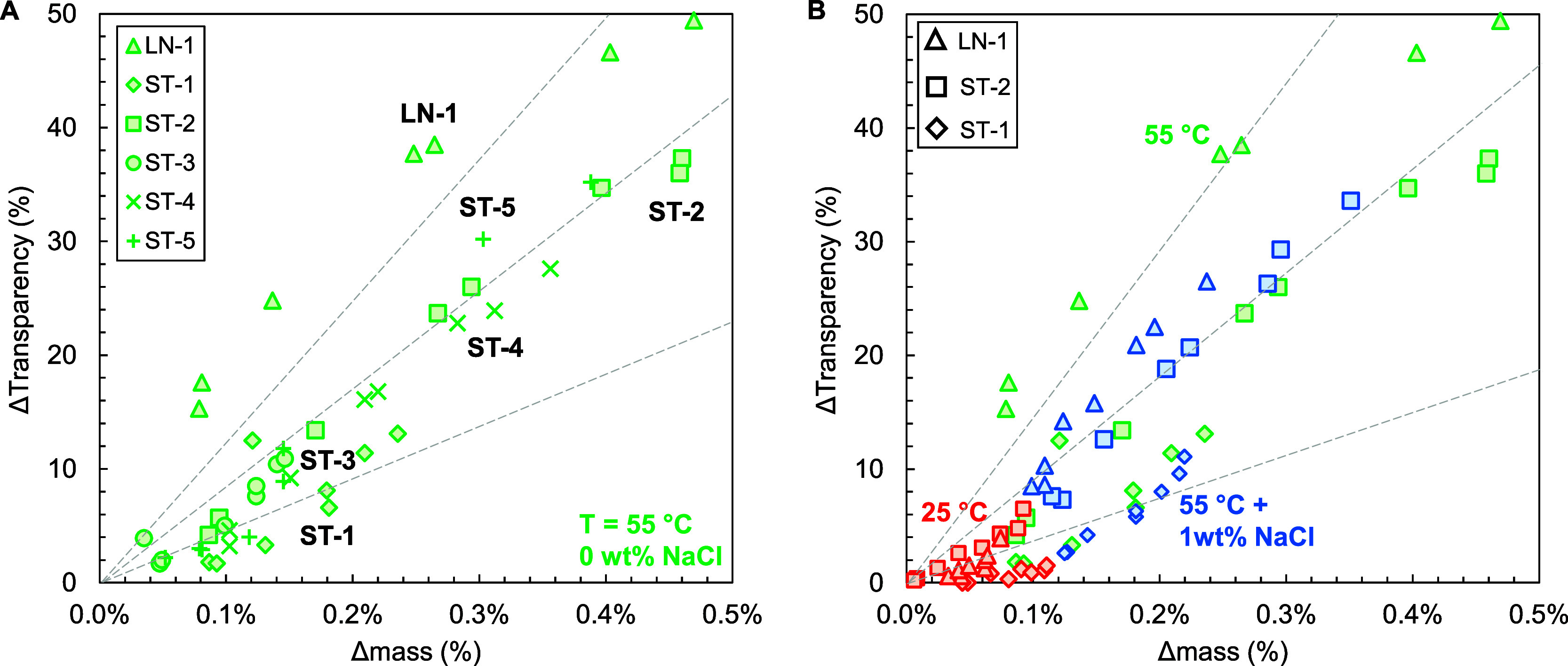
Loss of transparency as a function of relative mass increase for 2 mm optical plaques. The data presented in this figure are a carefully selected subset of the data presented in Figure 2. (A) Samples according to recipe LN-1, ST-1, ST-2, ST-3, ST-4, and ST-5 immersed in 55 °C demineralized water (without NaCl). (B) Samples according to recipe LN-1, ST-1, and ST-2 submerged in 25 and 55 °C demineralized water and 55 °C demineralized water with 1 wt % of NaCl.

Furthermore, it is noteworthy that for the same degree of absorption, the loss of transparency increases with an increasing soft phase volume. This could be an effect of the composition of the soft phase or, similar to the hypothesis mentioned above, an effect of the BCP morphology. If this effect was caused by the soft phase composition, one would expect an increase of transparency loss with the butadiene content. An increased butadiene content makes the soft phase less dense and more mobile and thus more susceptible to large water clusters that distort the polymer matrix. As ST-2 and ST-5 have a significantly higher butadiene content in the soft phase (58% and 63%, respectively) compared to LN-1 (50%), the difference in transparency loss for the same level of water absorption is indicative of a morphological effect on water clustering.

Figure 3B shows that the relation between transparency loss and water absorption for each polymer structure is independent of the temperature. For each of the samples, the 25 and 55 °C measurements seem to fit to the same S-curve. For ST-1 and ST-2, this relation is also valid once the samples are immersed in salty demineralized water, consistent with the hypothesis of a purely osmotic effect. However, in case of LN-1, the measurements in salt water deviate from the “normal” *S*-curve and the loss of transparency becomes significantly lower for the same amount of absorbed water. This implies either that NaCl has some mechanistic effect, or that it migrates into the material which would explain the relative mass increase for the same amount of absorbed water.

The reversibility of the transparency loss was investigated by placing the samples that were exposed to 55 °C in demineralized water for 360 h in a vacuum oven at 55 °C and 50 mbar for 72 h. The results of the transparency and mass recovery experiments are shown in Figure S7A,B, respectively. The transparency and mass of the samples quickly stabilized after a few hours in the vacuum oven and showed a residual transparency loss between 0.3% and 10%. Interestingly, the relative mass increase reduced to almost 0% and, for ST-3, ST-1, and ST-5, even below 0%. The negative values are indicative of evaporation of residual solvent (cyclohexane), which is more volatile than water. It is worth mentioning that in commercial polymers, traces of the residual solvent are unavoidable. To study the effect of water on polymer samples for daily life, the polymers have not been reprocessed or treated under high vacuum or with additional temperature protocols. The residual transparency loss could therefore be the result of water clusters that remain in the material, even after exposure to a high vacuum. Alternatively, it is conceivable that water caused permanent local distortion of the polymer nanostructure, which is unable to relax when water diffuses out of the local environment.

### 3.3 Effect of Temperature

The effect of temperature was further investigated by measuring the loss of transparency and mass increase through water absorption (after 336 h) at different temperatures for different polymer materials. Figure 4A demonstrates that, even at low temperature, each of the samples shows significant levels of absorption. The water uptake follows an exponential temperature dependence which is evident given the general temperature dependence of absorption and diffusion coefficients. Surprising, however, is that the second highest degree of water absorption is obtained by ST-1, without showing any transparency loss below 55 °C (Figure 4B). All other samples show a much closer alignment of mass increase and transparency loss. To understand this difference, the polymer structures have to be considered. Polymers ST-2, ST-3, and LN-1 each have a stable, well-defined soft and hard phase with glass transition temperatures significantly below the studied range. As a result, the morphology and total soft phase volume of each of those polymers are expected to be rather constant between 25 and 55 °C with no drastic shift between soft and hard phases or in the average segmental mobility. ST-1, on the other hand, is a polymer in which styrene and butadiene are statistically mixed in one uniform block. Therefore, the polymer consists of a single, uniform hard or soft phase depending on the temperature. The theoretical glass transition temperature is 61 °C, the polymer is expected to be glassy under the investigated conditions (≤55 °C), and, as a result, clustering should be limited to cavities and defects. Compared to pure PS, the material can absorb significantly more water due to the presence of butadiene making the overall phase less dense.

**Figure 4:**
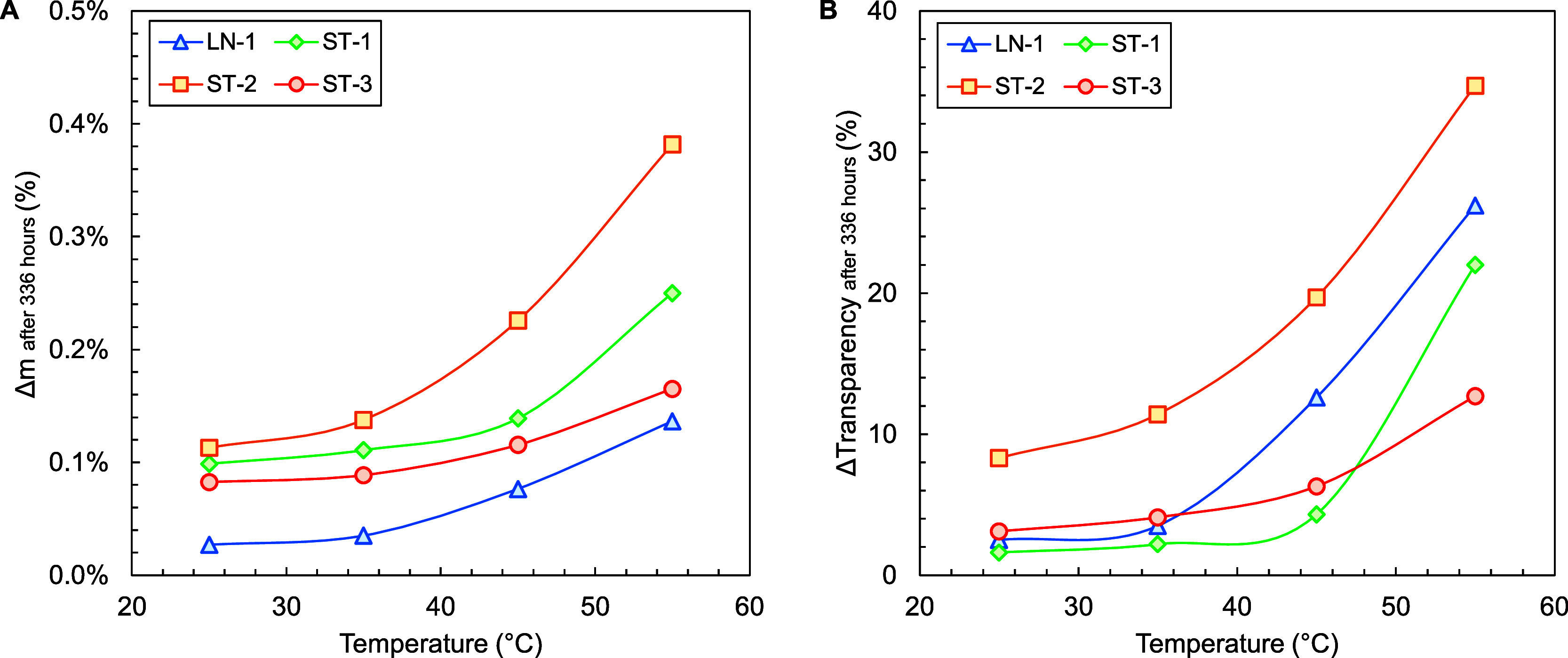
Relative mass increase (A) and loss of transparency (B) as a function of temperature for 2 mm optical plaques of SBC polymers according to recipes ST-1, ST-2, ST-3, and LN-1 submerged in demineralized water (with 0.9 wt % of NaCl) for 336 h.

To understand why the material suddenly loses transparency at 55 °C, a DMA measurement was performed on the sample before exposure to water (Figure S9). The measured glass transition temperature is around 70 °C but the glass-to-rubber transition starts around 50–55 °C. It is therefore expected that this sudden increase in transparency loss is the result of clusters being formed throughout the whole material as the polymer matrix loses its rigidity and gradually gains segmental mobility. Additionally, one could expect this temperature window to potentially decrease as a result of water-induced plasticization. A series of follow-up MDSC measurements showed, however, that the glass transition temperature only slightly decreases: from 68.1 °C prior to submersion to 67.1 and 66.1 °C after submersion in 55 °C demineralized water for 7 and 10 days, respectively (see Figures S10–S12).

### 3.4 Effect of Soft Phase Volume and Block Structure

Earlier measurements suggest a significant correlation between the volume of the soft phase and the extent of water-induced swelling, accompanied by an increased transparency loss with an increased soft phase volume. To gain more detailed insight into this relationship, a series of linear block copolymers were synthesized with an identical butadiene content but with varying block structures (Table 2, LN-1 to LN-7). The target is to exclude any effect related to the polymer composition and/or the termination/coupling approach. Using this approach, the only varying parameters are the soft phase volume (calculated in Table S2) and its composition (S/B ratio).

The materials were immersed in 55 °C salt-free demineralized water for 2616 h during which loss of transparency was measured at predefined exposure times. The loss of transparency as a function of the theoretical soft phase volume for different timestamps is shown in Figure 5. A clear relation between theoretical soft phase volume and loss of transparency (after a fixed exposure time) emerges that seems to follow an *S*-shaped curve for each timestamp. At soft phase volumes around 34 wt %, the samples initially exhibit a cloudy pattern similar to PS. However, after prolonged exposure, they start to lose their partial transparency and gradually become more opaque. Only when the soft phase volume surpasses 50 wt %, the samples rapidly turn opaque, losing a substantial amount of their transparency within just a few hundred hours. When the volume of the soft phase exceeds 60 wt %, the impact on transparency loss plateaus. Increasing the soft phase beyond this threshold does not result in a significant increase in the transparency loss. In this case, water absorption by the polymer sample could be the limiting step (compared to diffusion) in the formation of water clusters, thus hampering further increases in both extent and rate of transparency loss. Interestingly, for the measured structures, this plateau starts where a continuous soft phase is expected in the polymer morphology—at around 60% of soft phase in case of triblock copolymers.^26^

**Figure 5:**
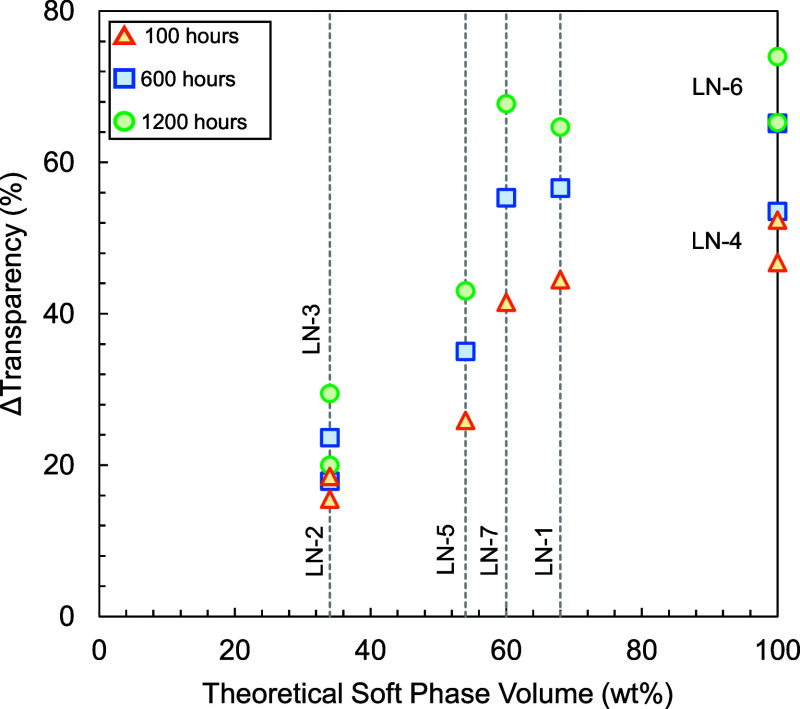
Loss of transparency as a function of theoretical soft phase volumes measured by immersing 2 mm optical plaques of polymeric materials according to recipe LN-1 to LN-7 (Table 2) in salt-free 55 °C demineralized water and measuring transparency after 100, 600, and 1200 h. The theoretical soft phase volume was determined based on the theoretical glass transition temperatures (see Table S2).

There is also a significant difference within the two sets of samples with identical theoretical soft phase volume. In the first case (LN-2 and LN-3), the copolymers are constructed based on pure styrene (hard phase) and pure butadiene (soft phase) blocks, and the difference is in the number of blocks: 3 in the case of LN-2 (S–B–S) and 5 in the case of LN-3 (S–B–S–B–S). In the second case (LN-4 and LN-6), the copolymers are constructed based on a styrene-rich block (soft phase 1) and a butadiene-rich block (soft phase 2) and the difference is again in the number of blocks. In both cases, the pentablock copolymers show a higher loss of transparency, and the difference increases over time. The absolute difference is also significantly larger for the high soft phase volume case (LN-4/LN-6) than that for the low soft phase volume case (LN-2/LN-3).

To rationalize the sudden increase in loss of transparency from LN-5 to LN-7 and gain more insight into why materials with the same theoretical soft phase volume have different losses of transparency, the sample morphologies were investigated by TEM imaging (Figure 6 and SI section S9) and SAXS measurements (SI section S9). The S–B–S triblock copolymer sample, LN-2, exhibits a bicontinuous structure without long-range order with a tendency toward a hexagonal structure (Figures 6A, S15, and S22A). This is consistent with the general phase diagram for S–B–S triblock copolymers, which predicts that PB cylinders in a PS matrix are expected for S–B–S triblock copolymers with a styrene content between 65 and 85 wt %.^26^ Moreover, cylindrical morphologies have been reported for analogous linear S–B–S products with a 74 wt % styrene.^27,28^ In contrast, the S–(B–S)_2_ pentablock copolymer, LN-3, shows a more lamellar-like morphology (Figures 6B, S16, and S22B), even though LN-2 and LN-3 have the same theoretical soft phase volume. DMA measurements also reveal a slightly higher styrene content in the soft phase of the pentablock structure compared to the triblock structure with a soft phase glass transition temperature of −85.0 and −89.6 °C, respectively, determined from the first step in the storage modulus curve (see Figures S13 and S14). Using the Fox equation, it was calculated that the pentablock and triblock structures possess soft phase volumes of 40 and 38 wt %, respectively, assuming the butadiene content in the hard phase is negligible. However, this minor variation in soft phase volume does not explain the disproportionate change in transparency loss, indicating that a shift in morphology is the underlying cause.

**Figure 6:**
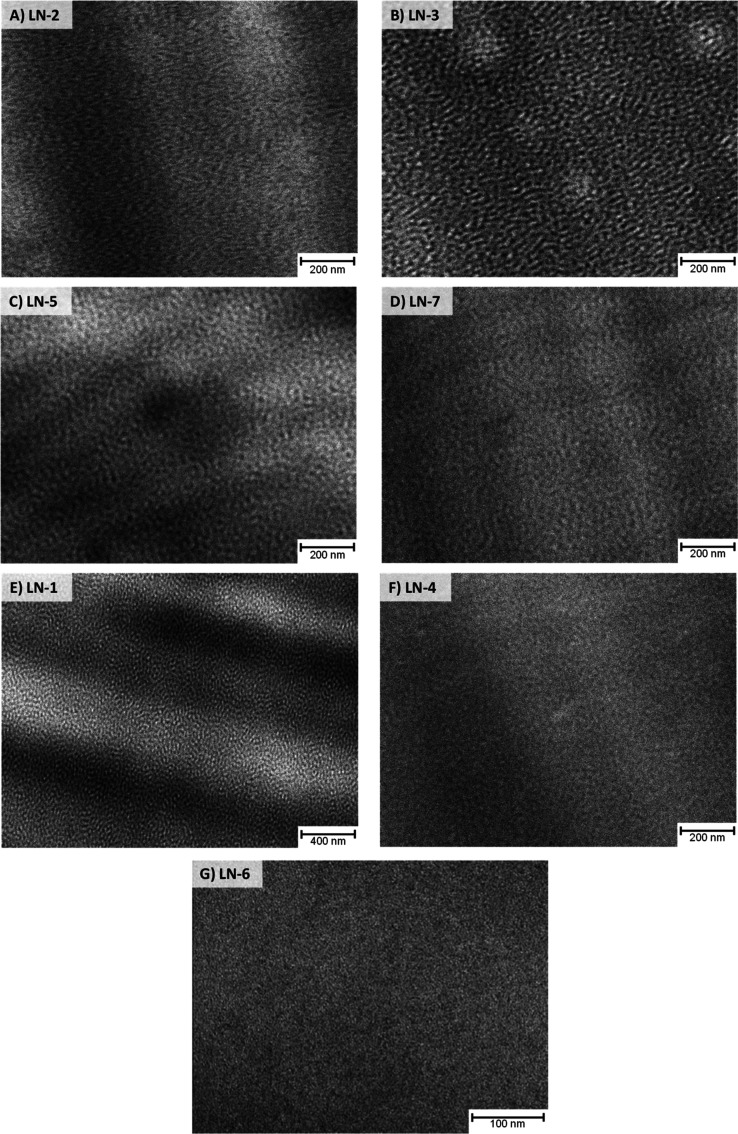
TEM images measured on the OsO_4_-stained microtome cut specimen produced from 2 mm optical plaques for: LN-2 (A), LN-3 (B), LN-5 (C), LN-7 (D), LN-1 (E), LN-4 (F), and LN-6 (G). The displayed magnification is chosen depending on the clarity of the observed nanostructure. Supplementary TEM images are provided in Figures S15–S21.

When the theoretical soft phase volume is further increased to 54 wt % by inflating the middle block of a triblock structure with styrene, LN-5, the lamellar phase seems to be maintained (Figures 6C and S17) and the loss of transparency shows a proportional increase. When the block structure is altered further and the theoretical soft phase volume is increased, two new morphologies arise. When the theoretical soft phase volume is increased to 60 wt % (34 wt % butadiene and 26 wt % styrene) and split into two identical blocks separated by an 8 wt % pure styrene block, LN-7, a bicontinuous phase without long-range order is obtained (Figures 6D, S18, and S22C). For star polymers with a similar structure, i.e., randomized styrene/butadiene blocks and a PS core, the gyroid morphology typically emerges.^29^ The change in the morphology is accompanied by a substantial and disproportionate increase in the loss of transparency (Figure 5), which is indicative of a strong role of the morphology. When the soft phase volume is increased to 68 wt % but kept as a single middle block, LN-1, a hexagonal morphology where hard cylinders are embedded in a soft matrix emerges (Figures 6E, S19, and S22D). Interestingly, the loss of transparency is at a similar level for LN-1 and LN-7, with LN-7 losing more transparency at high exposure times.

Finally, the theoretical soft phase volume was further increased to 100 wt % by shifting a fraction of the butadiene from the butadiene-rich phase directly into the styrene-rich phase and targeting a glass transition temperature below room temperature. In the case of a triblock structure, LN-4, this still resulted in mild phase separation where styrene-rich spheres are dispersed in a butadiene-rich phase (Figures 6F, S20, and S22E). For the pentablock variant, no phase separation occurred, and a uniform monophase was observed (Figures 6G and S21). The occurrence of mild phase separation in LN-4 and the absence of phase separation in LN-6—which happens once the chemical structure of different blocks are similar—could explain the difference in transparency loss, again indicating some morphological effects. Phase separation into a more rigid and a less rigid phase in LN-4 could, to a certain extent, limit the mobility of penetrant molecules compared to the case of a uniform phase, as is the case for LN-6.

Combining the measurements in Figures 5 and 6 with the previous analyses in Figures 1 and 4, it can be concluded that the soft phase is predominantly responsible for the extent to which the material loses its transparency. This is consistent with previous findings that show that clustering in polymers above and below their glass transition temperatures is drastically different. In glassy polymers, water diffuses through the matrix into cavities where it clusters together, which can cause a hazy, mist-like pattern. In polymers below their glass transition temperature, however, clusters can form anywhere due to their high segmental mobility. Here, the glass transition temperature should be viewed less as a direct predictor of transparency loss and more as a threshold indicating whether a phase can be significantly deformed by water cluster formation. When the *T*_g_ of a phase is above the process temperature, water clusters may still form, but the rigid matrix restricts their growth to below the wavelength of visible light, resulting in limited scattering. In contrast, if the *T*_g_ is below the process temperature, then the phase exhibits increased segmental mobility, enabling the growth of larger clusters and substantial local deformation and scattering. Table S2 supports this interpretation by identifying which phases are likely to experience such distortions and providing estimates of the corresponding soft phase volumes, thereby offering a clearer picture of the extent of potential transparency loss. The findings shown above indicate that they can be combined with a general theory for block copolymers. Water is absorbed by the BCP and can subsequently diffuse both in and through the hard and soft phases of BCPs. In the hard phase, water will only cluster in preexisting cavities and defects, while in the soft phase of the material, it can cluster anywhere. This theory also implicitly imposes a strong effect of the morphology of the BCP that explains why the loss of transparency does not simply increase proportionally to the soft phase volume (Figure 5). Consider, for example, a cylindrical morphology with soft cylinders dispersed in a hard matrix, as is the case, for example, for S–B–S triblock copolymers with a butadiene content between 15% and 35% for example.^26^ In this morphology, clusters can freely grow in the soft cylinders, as well as in voids/defects in the matrix. As the water clusters grow in size, however, they will push against the glassy matrix, which they are unable to deform. The result of water absorption will therefore be small clusters dispersed throughout the soft cylinders, which can only lead to a limited drop in transparency. Alternatively, consider a morphology with a continuous soft phase, as is the case, for example, for product LN-1 where hard cylinders are dispersed in a soft matrix. In this morphology, water clusters have more freedom to grow, leading to increased quantity and size, which can be attributed to the larger volume of the soft phase and the enhanced mobility of the surrounding matrix.

### 3.5 Predictive Modeling

Throughout the experiments, the loss of transparency always seemed to reach a plateau; however, it was difficult to predict an asymptotic limit value. Long-term measurements showed that even after 2616 h, the loss of transparency keeps increasing. This hampers product development in which a specific optical performance is targeted for prolonged water exposure. To address this, the long-term measurements (2616 h) of polymer LN-1 to LN-7 were used to assess two modeling strategies. The first goal was to determine how much measurement time would be required to make an accurate estimate of the long-term performance. The second goal was to evaluate whether machine learning techniques can be applied to predict long-term optical performance of SBC in water prior to synthesis of the material.

#### 3.5.1 Predictive Modeling through Regression-Extrapolation

In the first modeling approach, a classical regression-extrapolation approach was investigated. The main goal here was to identify an adequate mathematical description and to assess how much measurement time is required to accurately predict the long-term optical performance. For this purpose, different mathematical functions were evaluated that describe a decay-like behavior. Based on mathematical intuition, a space of candidate functions was defined (Table S3). Through initial assessment, the details of which are provided in SI section S10.1, the candidate space was reduced to three descriptions (see Table 3): a power law, a first-degree exponential function, and a second order rational function.

The three candidate functions were subsequently used in a series of demonstrating experiments in which the target was to predict the optical performance after 2616 h of exposure to 55 °C salt-free water. For this purpose, the polymer products LN-1 to LN-7 were immersed in a water bath, and the loss of transparency was measured at predetermined timestamps over the period of 2616 h. A custom Python script was applied to methodically incorporate each measurement point into the data set. With each addition, a regression analysis was performed for each of the three candidate functions to predict optical performance at 2616 h, comparing these predictions to the measured values. Additionally, the fit of each candidate model at a specific measurement time was assessed by using a statistical F-test for validation.

Figure 7 shows an example of the approach described above applied to polymer LN-1. Below 200 h of measurement time, the power law description severely overestimates the transparency loss. This error quickly decreases and from 500 and 600 h onward, the absolute prediction error, i.e., the difference in loss of transparency between measured and calculated values decreases below 10 and 5%, respectively (Figure 7A). The prediction error in the rational and exponential descriptions decreases notably slower, and both require a measurement time between 1500 and 2000 h to predict the performance at 2616 h. Figure 7B indicates that at 600 h of measurement time, both rational and exponential functions show a fast plateau and treat the last points as a sort of overshoot. Similar behavior of the functions was observed at other snapshots during the measurements (Figure S23A,C,D). A statistical *F*-test also showed that the exponential and rational descriptions provide no significant improvement in fit compared to the simpler power law model after 500 h of measurement.

**Figure 7:**
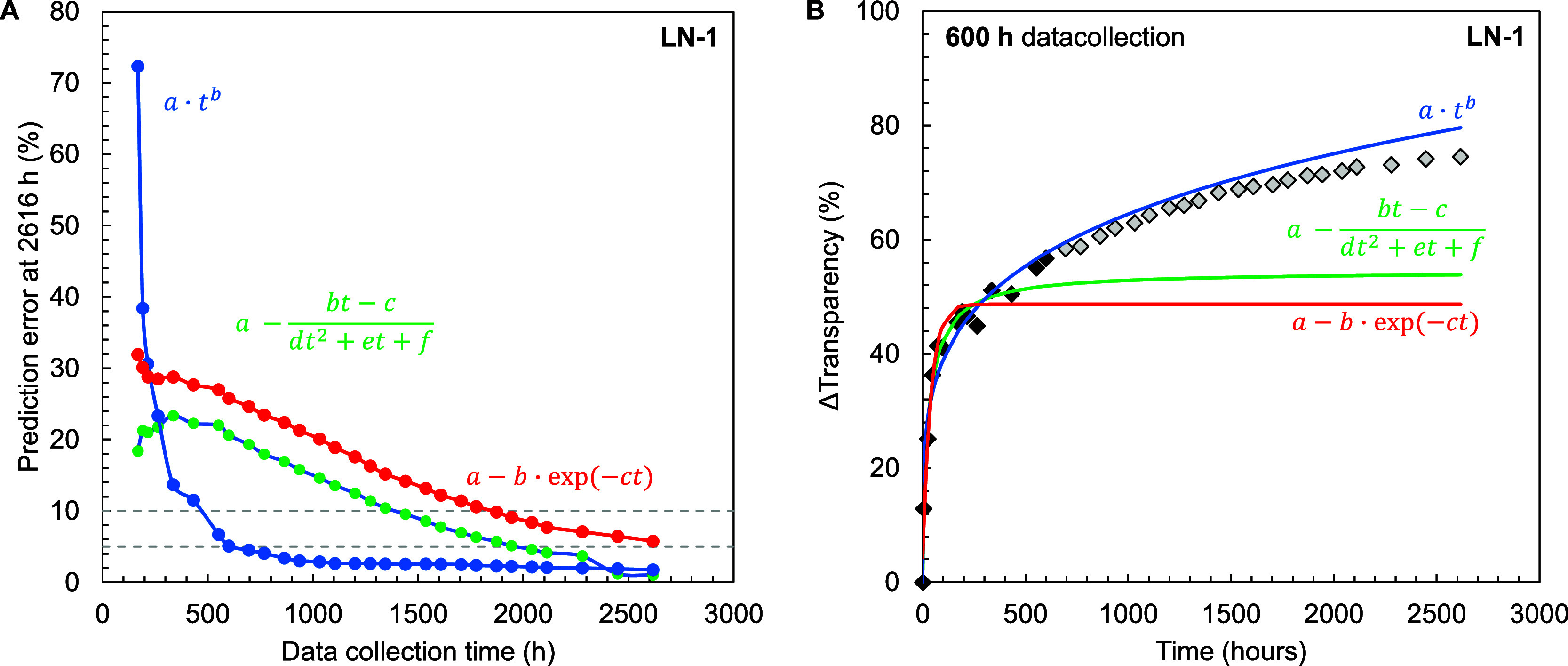
Long-term optical performance experiment for polymer LN-1 in 55 °C salt-free demineralized water: (A) absolute prediction error between the extrapolated value calculated with the regressed function based on a data subset from 0 until the data collection time with a comparison between the three candidate functions. The dashed lines represent, respectively, 10% and 5% absolute prediction error. An (B) illustration of the regression-extrapolation performance of the three candidate systems using 600 h of measurement data and comparing to 2616 h of measured data.

The generality of these findings was demonstrated by applying the same strategy to polymer materials LN-2 to LN-7, see Figures S24–S31. A total measurement time of around 600 h was generally sufficient to provide an accurate prediction for the optical performance at 2600 h within an absolute prediction error of 5%.

#### 3.5.2 Application of an Artificial Neural Network

While the method described above already shortens the measurement time required to quantify long-term optical performance of SBCs in a high water activity environment, it does not provide any further insight in the overall mechanism, nor does it allow one to estimate the performance prior to synthesis and/or testing of the material. Therefore, a more comprehensive approach is desired. In this section, it is investigated whether an artificial neural network is appropriate to predict the long-term optical performance of SBC polymers in water at elevated temperature. The soft phase volume was used as a descriptor, and the model was trained using the long-term measurement data of structures LN-1 to LN-7 (for data, see Figures S24–S30) in salt-free demineralized water at 55 °C. The model was used to construct a transparency loss diagram and subsequently tested against measurement data that were not included in the training set, see Figure 8. The model predicts that at low soft phase volume, the loss of transparency increases fastest with increasing soft phase volume. The effect of exposure time, however, is rather small below 40% of the soft phase. Measurement data of polymer ST-1 suggest, however, that the model overestimates the loss of transparency at low soft phase volume. As the training range only extends between 35 and 100 wt % of soft phase volume, it is not surprising to expect poor performance below 35% and additional measurements would be required to more accurately describe that region.

**Figure 8:**
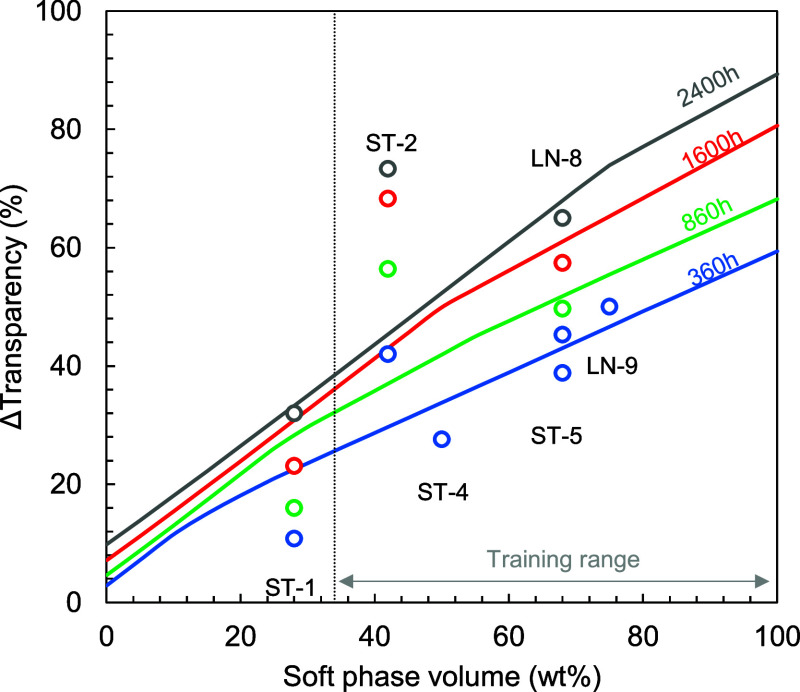
Loss of transparency as a function of the soft phase volume for different exposure times to a salt-free demineralized water environment at 55 °C as predicted with an artificial neural network. The model was trained using the 2616 h of measurement data obtained for polymers LN-1 to LN-7 and then tested by comparing to measurement data obtained for polymer ST-1, ST-2, ST-4, ST-5, and LN-9.

After a certain point, which depends on the exposure time, the loss of transparency increases slower with the soft phase volume. This corresponds to the right side of the S-shaped curve, which is observed in Figure 5. By using data not included in the model, a generally good fit between the model and the investigated structures could be shown. The fit with data from LN-8 is not surprising, given that the only difference lies in a slightly higher butadiene content. Interestingly, when butadiene was replaced by isoprene as conjugated diene in a triblock copolymer, the model retained adequate predictability (LN-9). The measured loss of transparency in polymer materials ST-4 and ST-5 follow the general trend but were already substantially overestimated by the model. Polymer ST-2, on the other hand, completely breaks the simulated trend as well as the general behavior observed in Figure 5 with a transparency loss that far exceeds structures with a significantly higher soft phase volume. This once again shows that the loss of transparency is not simply proportional to the soft phase volume and that the disparity between soft phase volume and transparency loss indicates a strong effect of the block copolymer morphology on the extent that water clusters can grow. The hypothesis of morphological effects is further strengthened by the fact that the ST-2 structure does not follow the rule of transition from soft spheres to soft cylinders, to lamella, to hard cylinders, to hard spheres as is observed in S–B–S triblock copolymers. It is generally known that this particular star-shaped polymer structure shows a co-continuous gyroid morphology.^27^

Figure 8 demonstrates that computer modeling, particularly machine learning-based models, could be a useful tool in the design of polymers with high optical performance in water environments. However, the current demonstrator model needs to be completed with data that are more evenly distributed along the soft phase volume space. In addition, it is expected that the inclusion of a morphological factor as well as soft phase composition will greatly improve the adequacy of the model. Moreover, the incorporation of the temperature and NaCl concentration will also increase the versatility of the model.

## 4 Conclusions

Water-induced transparency loss in SBCs has been studied under a variety of exposure conditions. Similar to homopolymers, the loss of transparency that occurs when the polymer is exposed to water at elevated temperatures is expected to be the result of water clusters, distorting the surrounding polymer matrix and causing local changes in the refractive index. This hypothesis was further supported by comparing the performance of the standard material with that of a material without additives and a material in which the salt from the initiation step has been almost completely washed out. Both omissions were found to have little effect on the overall optical performance in water at elevated temperatures.

Subsequently, a range of different linear and star-shaped polymers were synthesized and immersed under different conditions. A strong effect of temperature, the presence of NaCl, and soft phase volume was observed. The effect of temperature generally proved to be determining for diffusion and absorption, showing the expected exponential dependencies. However, when the investigated temperature started to come close to the material glass transition temperature, a drastic loss of transparency is observed. Transparency loss below the glass-to-rubber transition is small. The material surface shows a hazy, mist-like pattern. When the temperature is increased into the glass-to-rubber transition region, however, the material quickly starts to lose its transparency.

To gain a better understanding of the role of the soft phase volume and morphology, a series of linear polymers were synthesized, each containing an identical butadiene content but a different soft phase volume. A strong correlation between soft phase volume and loss of transparency clearly emerged, which, together with the effect of temperature, shows that the loss of transparency is mainly caused by the soft phase volume. As a result, one can in principle describe water absorption in block copolymers as the sum of the behavior in the hard and soft phase. Water will, on the one hand, diffuse through the hard phase and enter preexisting cavities where it can form clusters without drastically altering the transparency of the material. On the other hand, water can form clusters anywhere in the soft phase. Because of the segmental mobility, the soft phase will grow substantially in size and cause local changes in the refractive index throughout the material. It was indicated that this loss of transparency is not simply proportional to the soft phase volume. Discrete jumps are observed, and some recipes with a lower soft phase volume do show a higher loss of transparency, indicating an impact of the block copolymer morphology. When the block copolymer has a continuous hard phase, diffusion through the hard phase will limit the rate at which water molecules reach the soft phase segments. In addition, the growth of clusters in the soft phase segments will be hampered by the surrounding glassy matrix. When the soft phase is continuous, however, the situation changes and the soft phase is significantly less limited by the presence of a hard phase. This interplay between soft and hard phase is specifically interesting in the case of a cocontinuous gyroid phase, which shows a substantially higher transparency loss after prolonged water exposure than polymers with a higher soft phase volume.

Finally, different methods for predictive modeling were investigated. It was observed that a power law description is adequate in describing time-dependent loss of transparency and that 600 h of measurement time allows accurate prediction of the optical performance up to at least 3 months. Alternatively, it was demonstrated that an artificial neural network could provide guidance prior to SBC synthesis. However, the constructed model is merely a demonstrator and should be extended by including training data with a better distribution along the soft phase volume space, an additional descriptor to account for the soft phase composition(s), and a descriptor to account for morphological effects, through a categorical value for example.

## Supplementary Material



## Notes

Additional polymer characterization results as well as experimental setup, construction of the artificial neutral network, details of the washing procedure, pictures of the initial optical measurements, calculation of the theoretical soft phase volumes, reversibility tests, DSC and DMA measurements, TEM images and SAXS measurements, and Regression-extrapolation (PDF)
