# Supplementary material for: Water-Induced Transparency Loss in Styrene Butadiene Block Copolymers: Mechanism, Morphology, and Predictive Modeling
Source: Macromolecules. 2025 Jul 30;58(15):7673–85. doi: 10.1021/acs.macromol.5c01354 (PMC12356056; doi:10.1021/acs.macromol.5c01354)
Supplement: Supplementary file 1 [file ma5c01354_si_001.pdf]

Supporting Information

Water-induced Transparency Loss in Styrene Butadiene Block Copolymers: Mechanism, Morphology and Predictive Modeling

Jenoff E. De Vrieze [1,], Michiel Verswyvel [1], Kinza Y. Ghulam [2], Bart-Jan Niebuur [3], Tobias Kraus [3,4], Markus Gallei [2,5,*], Norbert Niessner [1]*

[1] INEOS Styrolution Group GmbH, Global R&D Laboratories, Mainzer Landstraße 50, 60325 Frankfurt, Germany.

[2] Saarland University, Polymer Chemistry, Campus C4 2, 66123 Saarbrücken, Germany.

[3] INM – Leibniz Institute for New Materials, Campus D2 2, 66123 Saarbrücken, Germany.

[4] Saarland University, Colloid and Interface Chemistry, Campus D2 2, 66123 Saarbrücken, Germany.

[5] Saarene, Saarland Center for Energy Materials and Sustainability, Campus C4 2, 66123 Saarbrücken, Germany.

* Corresponding authors.

Table of Contents

1. Polymer Characterization results 3
2. Experimental setup 5
3. Construction of the artificial neural network 5
4. Details of the washing procedure 6
5. Pictures of the initial optical measurements 7
6. Calculation of the theoretical soft phase volumes 9
7. Reversibility tests 9
8. DSC and DMA measurements 10
9. Supplementary TEM images and SAXS measurements 14
10. Regression-extrapolation 19
    10.1. First assessment. 19
    10.2. Demonstrating experiments 21

1. Polymer Characterization results

Formation of the chains

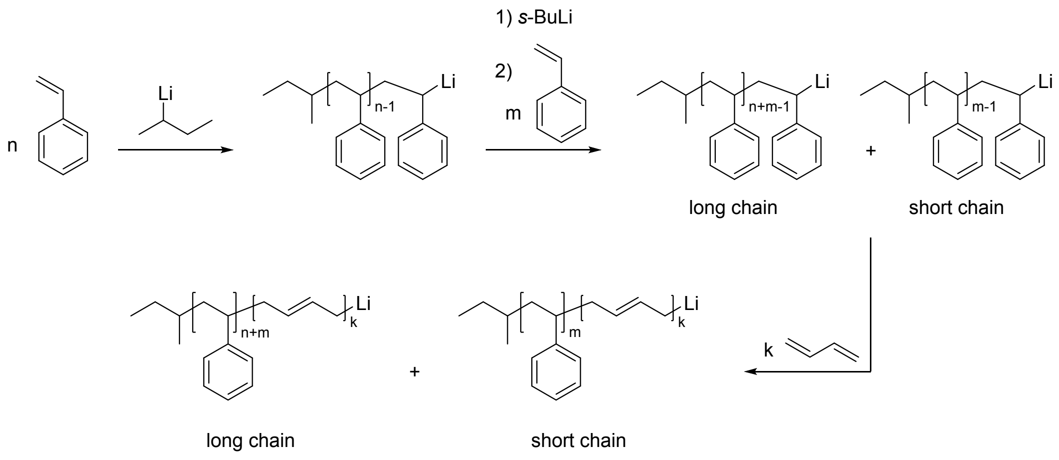

Coupling to stars

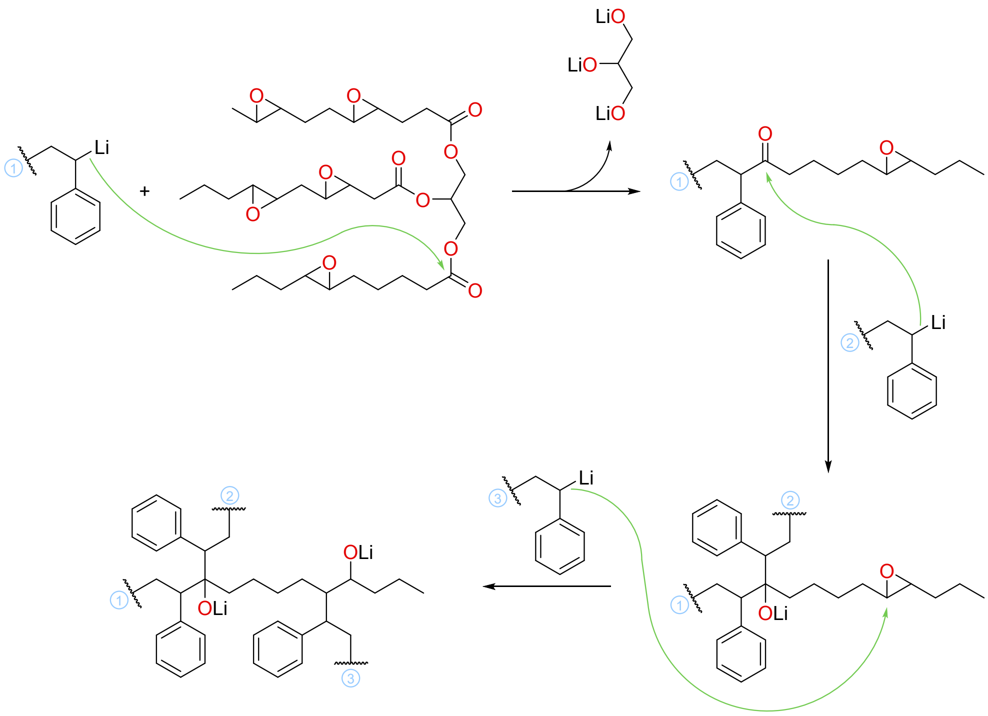

Scheme 1: Representative depiction of the synthesis, coupling and polymer structure of ST-3.

Table S1. Characterization results for the different synthesis recipes. M_{long}: molecular weight of the long block before coupling, D_{long}: polydispersity of the long chain before coupling, M_w: weight average molecular weight of the product after coupling (or termination), MVR melt volume rate measured at 200 °C with a 5 kg weight according to ISO 1133. All molecular weights are reported with respect to polystyrene standards.

Name	Structure	M_{long} kg/mol	D_{long} -	M_w kg/mol	MVR cm³/10min	$D_{coupled}$ -
ST-1	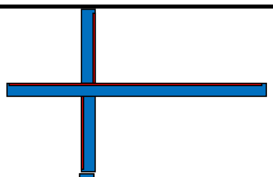	137	1.10	248	11	1.66
ST-2	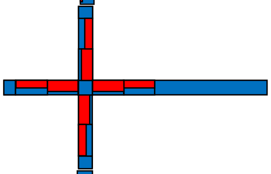	104	1.04	148	14	1.52
ST-3	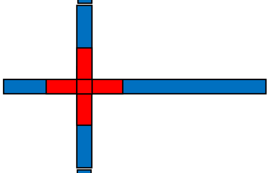	102	1.09	163	12	1.55
ST-4*	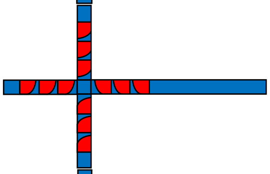	87	1.13	149	6	1.46
ST-5*	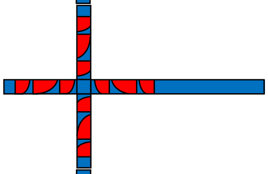	111	1.17	176	6	1.63
ST-6	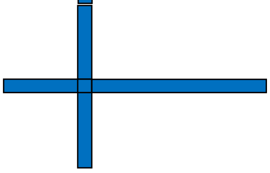	76	1.02	173	9	1.51
LN-1	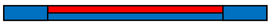	118	1.12	117	15	-
LN-2	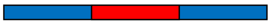	129	1.08	127	12	-
LN-3	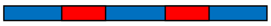	126	1.10	125	10	-
LN-4	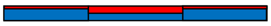	165	1.09	164	19	-
LN-5	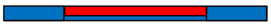	129	1.29	126	5	-
LN-6	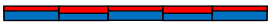	160	1.07	155	21	-
LN-7	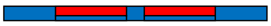	130	1.15	131	12	-
LN-8	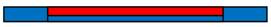	113	1.15	115	14	-
LN-9	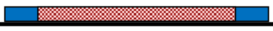	114	1.05	118	17	-

2. Experimental setup

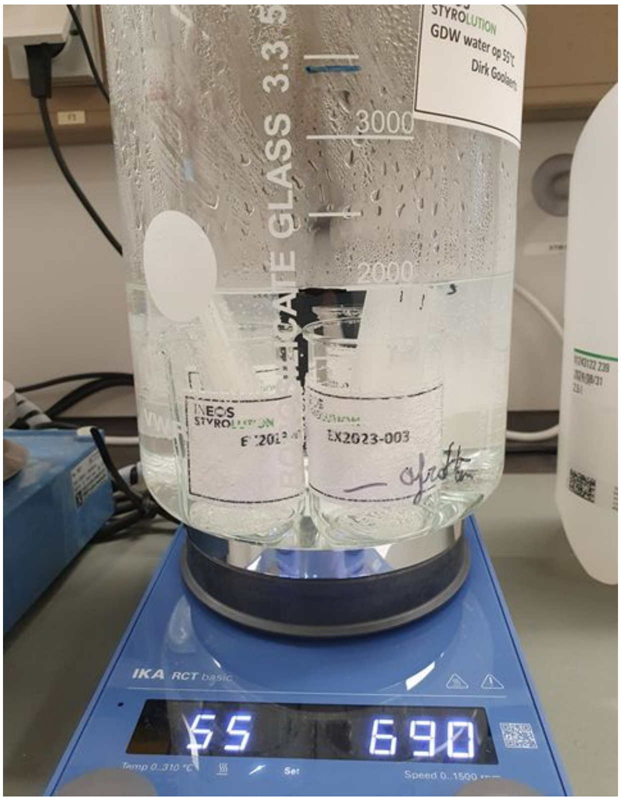

Figure S1. Example of the setup in which 2 mm optical plaques are exposed to a water environment at a set temperature (in this case 55 °C) and at a set NaCl concentration (in this case 0 wt%). The plaques are immersed in small beakers which are placed in a 5 l beaker. The water level is controlled in such a way that the plaques cannot leave the small beakers to float on the surface. The mixture is stirred at 690 rpm.

3. Construction of the artificial neural network

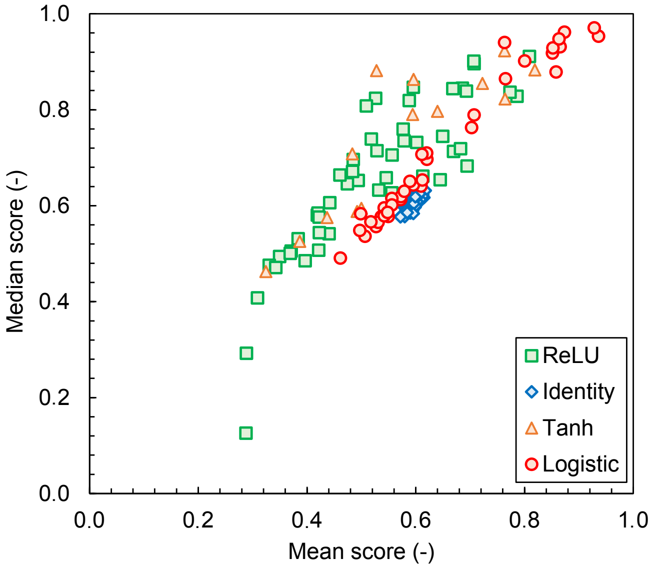

Figure S2. Determination of the appropriate neural network architecture by scanning different combinations of number of hidden layers, number of neurons in each layer and neuron activation function. Throughout the assessment, the lbfgs solver was used with a maximum of 10000 iterations and an L_2 regularization factor of 0.0001.

4. Details of the washing procedure

A modified version of LN-1 was produced. The achieve this, the following adapted recipe was applied:

1. Load the reactor with 15 kg of cyclohexane and heat to 50 °C.
2. Add 2 mL of diphenylethylene and titrate the solution dry with s-BuLi until color change.
3. Dose 3.26 g of a 5 wt% potassium tert-amylate (in cyclohexane) solution.
4. Dose 30.2 g of a 12 wt% s-BuLi (in cyclohexane) solution.
5. Dose 800 g of styrene and wait until all monomer is consumed, indicated by a peak in the temperature profile in the reaction medium.
6. Dose a mixture of 1700 g butadiene and 1700 g styrene and wait until all monomer is consumed, indicated by a peak in the temperature profile in the reaction medium.
7. Dose 800 g of styrene and wait until all monomer is consumed, indicated by a peak in the temperature profile in the reaction medium.
8. Terminate with by dosing 10 mL water and wait 15 min.
9. Dose 5 L water and stir for 15 min.
10. Turn of the stirrer and wait 2 hours.
11. Open the bottom valve and remove the water phase until organic phase is observed.
12. Extrude the organic phase as described and proceed to injection molding.

An ICP measurement revealed a concentration of 0.0107 mol/L present in the water. Given that nearly the full 5L of water was recovered after the liquid-liquid extraction, this corresponds to about 0.372 g of Lithium atoms, which is 94.7% of the added 0.393 g Li atoms.

5. Pictures of the initial optical measurements

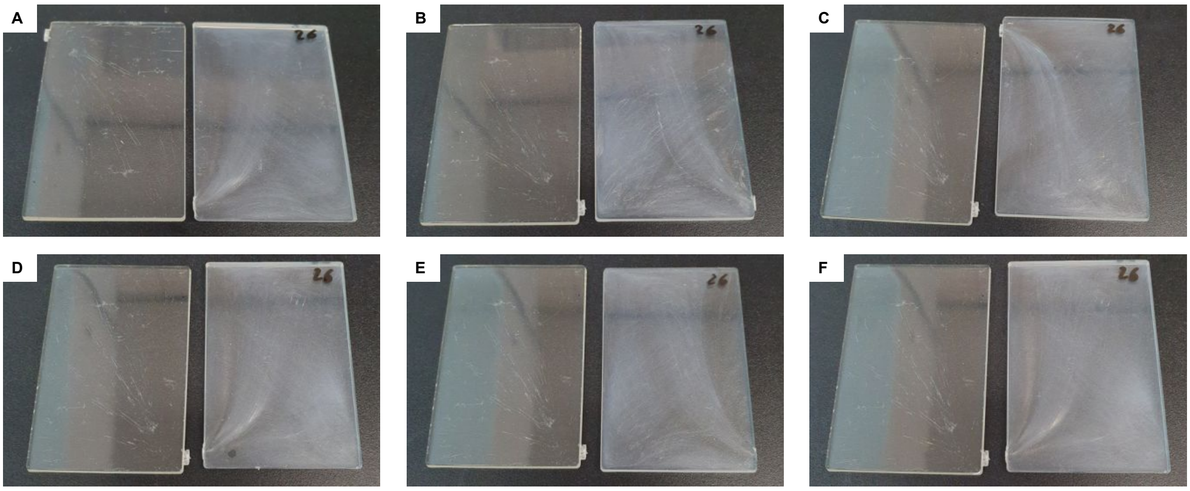

Figure S3. Pictures taken from injection molded 2 mm optical anionic polystyrene plates after exposure to 55 °C demineralized water for: (**A**) 8 hours, (**B**) 24 hours, (**C**) 48 hours, (**D**) 120 hours, (**E**) 171 hours and (**F**) 336 hours.

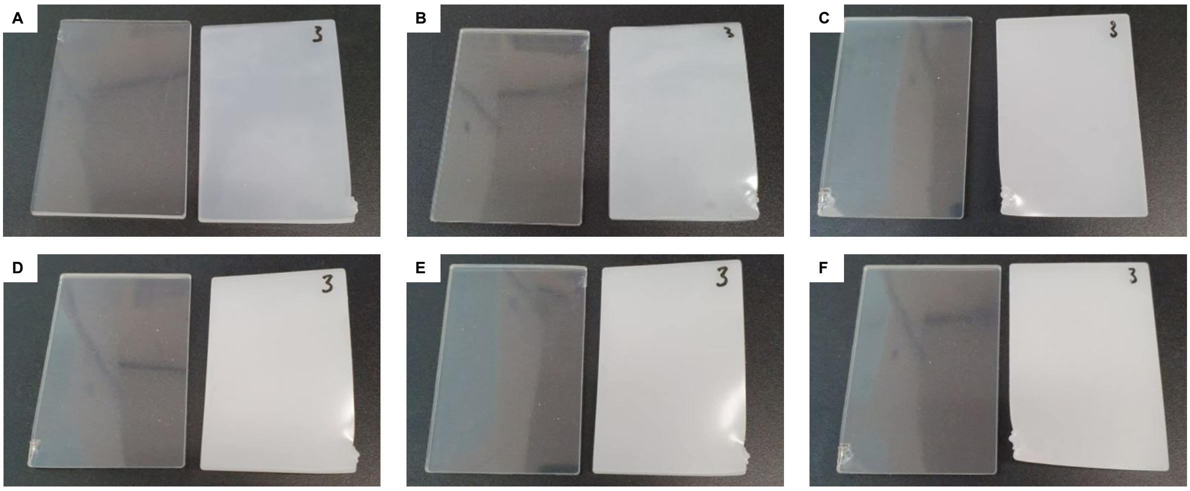

Figure S4. Pictures taken from injection molded 2 mm optical SBC (LN-1 recipe) plates after exposure to 55 °C demineralized water for: (**A**) 8 hours, (**B**) 24 hours, (**C**) 48 hours, (**D**) 120 hours, (**E**) 171 hours and (**F**) 336 hours.

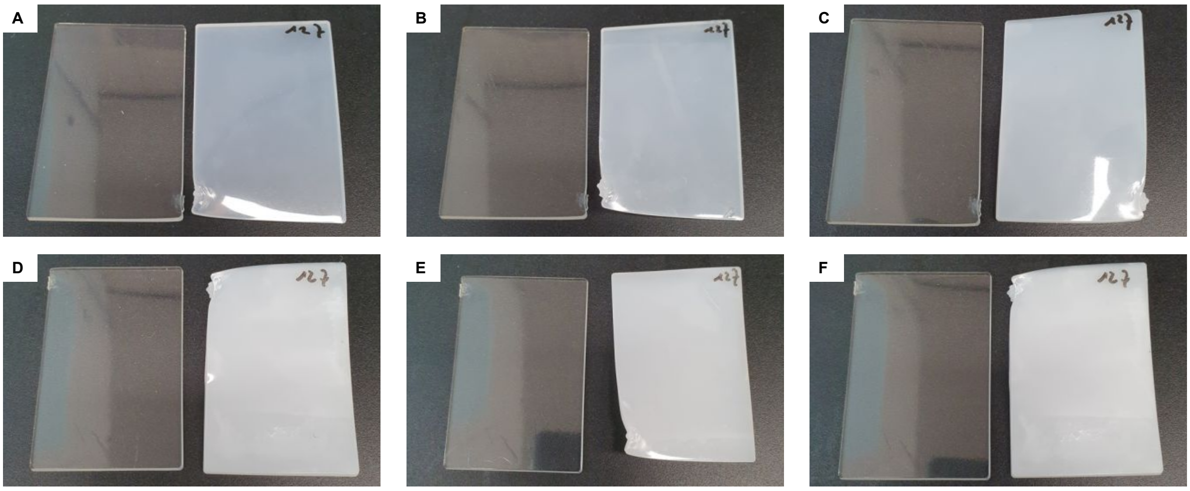

Figure S5. Pictures taken from injection molded 2 mm optical SBC (LN-1 Li-free recipe) plates after exposure to 55 °C demineralized water for: (**A**) 8 hours, (**B**) 24 hours, (**C**) 48 hours, (**D**) 120 hours, (**E**) 171 hours and (**F**) 336 hours.

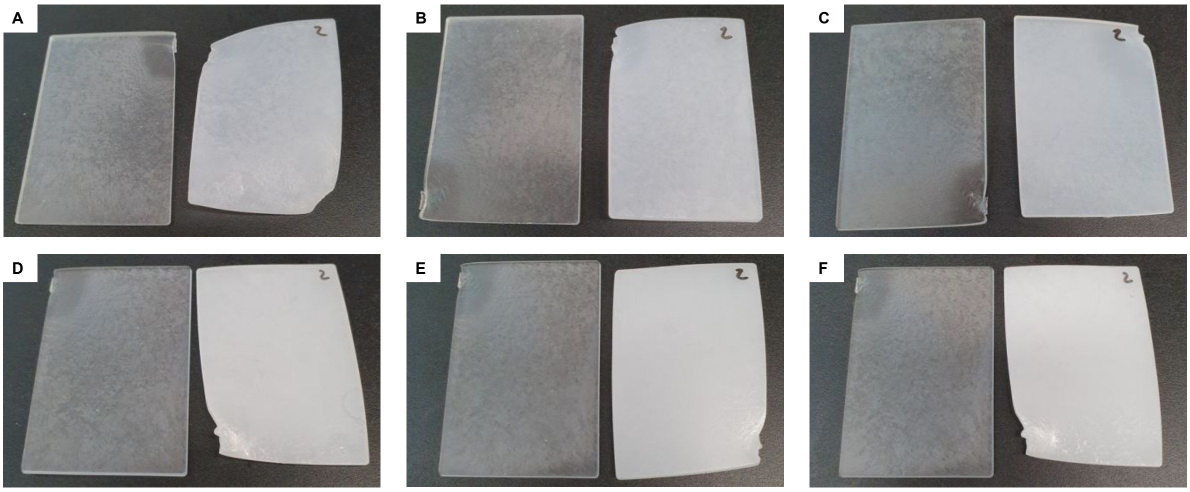

Figure S6. Pictures taken from injection molded 2 mm optical SBC (LN-1 stabilizer-free recipe) plates after exposure to 55 °C demineralized water for: (**A**) 8 hours, (**B**) 24 hours, (**C**) 48 hours, (**D**) 120 hours, (**E**) 171 hours and (**F**) 336 hours.

6. Calculation of the theoretical soft phase volumes

Table S2. Calculation of the soft phase volumes for each of the recipes in Table 1 using the Fox equation. When the glass transition temperature was calculated below the immersion temperature, the block was attributed to the soft phase. When the glass transition temperature was above the immersion temperature, the block was attributed to the hard phase.

Name	BD_{tot}	Block 1		Block 2		Block 3		Block 4		Block 5		V_{soft}
		x_{BD}	T_g	x_{BD}	T_g	x_{BD}	T_g	x_{BD}	T_g	x_{BD}	T_g	
	wt%	-	°C	-	°C	-	°C	-	°C	-	°C	wt%
ST-1	10	No phase separation (small S blocks): uniform phase of 10% BD (T_g = 61 °C)										0
ST-2	26	0.00	100	0.00	100	0.50	-37	0.61	-55		100	45
ST-3	25	0.00	100	0.00	100	1.00	-100		100		100	25
ST-4*	38	0.00	100	0.00	100	0.75	-73	0.76	-75	0.76	-75	50
ST-5*	38	0.00	100	0.00	100	0.58	-49	0.60	-53	0.76	-75	68
ST-6	0	0.00	100		100		100		100		100	0
LN-1	34	0.00	100	0.50	-37	0.00	100		100		100	68
LN-2	34	0.00	100	1.00	-100	0.00	100		100		100	34
LN-3	34	0.00	100	1.00	-100	0.00	100	1.00	-100	0.00	100	34
LN-4	34	0.25	16	0.50	-37	0.25	16		100		100	100
LN-5	34	0.00	100	0.63	-57	0.00	100		100		100	54
LN-6	34	0.25	17	0.50	-37	0.25	16	0.50	-37	0.25	17	100
LN-7	34	0.00	100	0.57	-48	0.00	100	0.57	-48	0.00	100	60

7. Reversibility tests

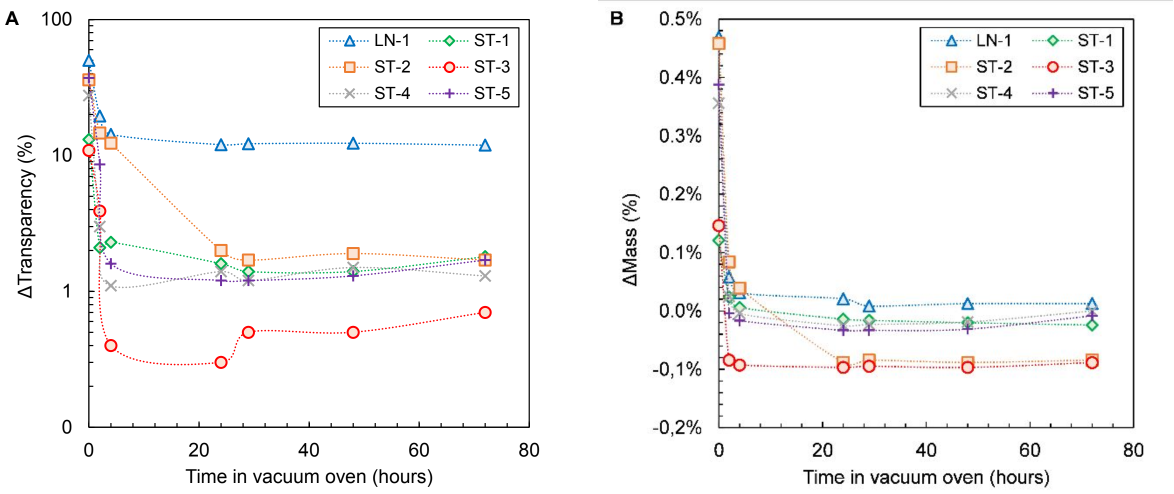

Figure S7. Reversibility test for 2 mm optical plaques that were immersed for 360 hours in 55 °C water and subsequently placed in a vacuum oven at 55 °C and 50 mbar. The plaques were removed for mass and transparency measurement after 2, 4, 24, 29, 48 and 72 hours.

8. DSC and DMA measurements

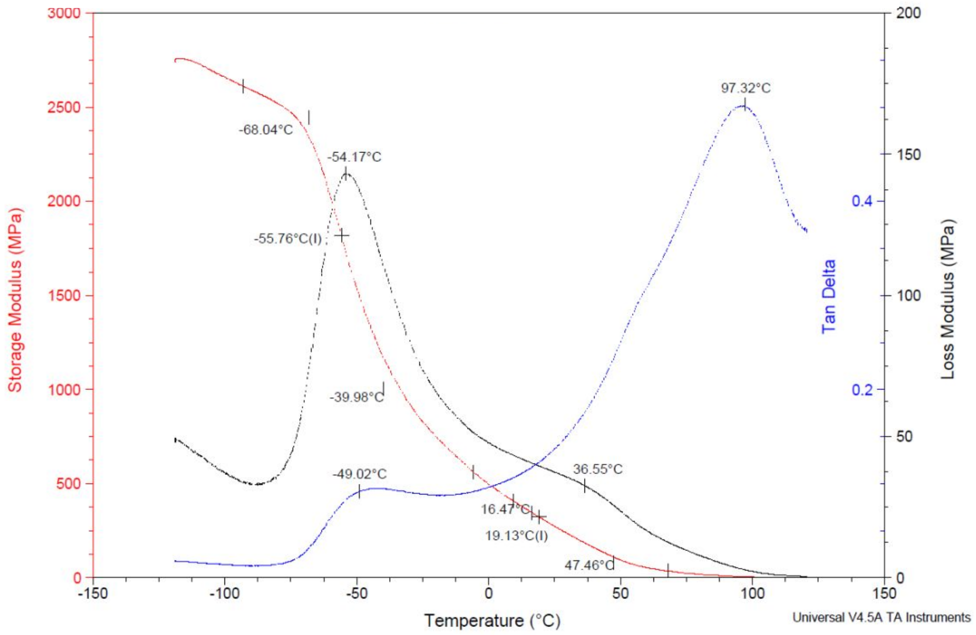

Figure S8. DMA measurement of the ST-5 sample starting at -120 °C for 1 min and heating to 120 °C with a ramp of 2 °C/min after which the sample is kept at 120 °C for 1 min. The measurement is performed with an amplitude of 25 µm and a frequency of 1 Hz.

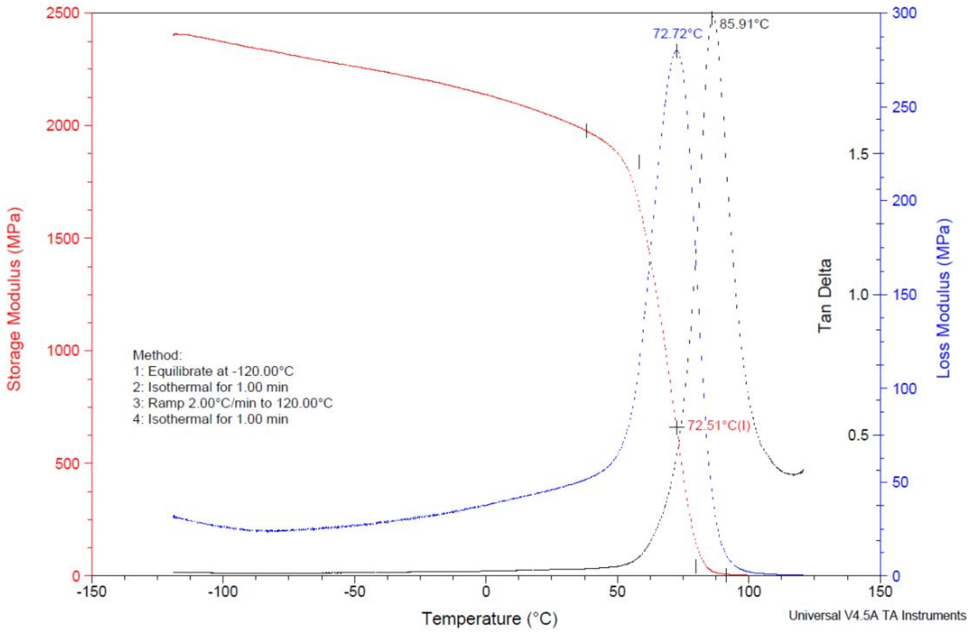

Figure S9. DMA measurement of the ST-1 sample starting at -120 °C for 1 min and heating to 120 °C with a ramp of 2 °C/min after which the sample is kept at 120 °C for 1 min. The measurement is performed with an amplitude of 25 µm and a frequency of 1 Hz.

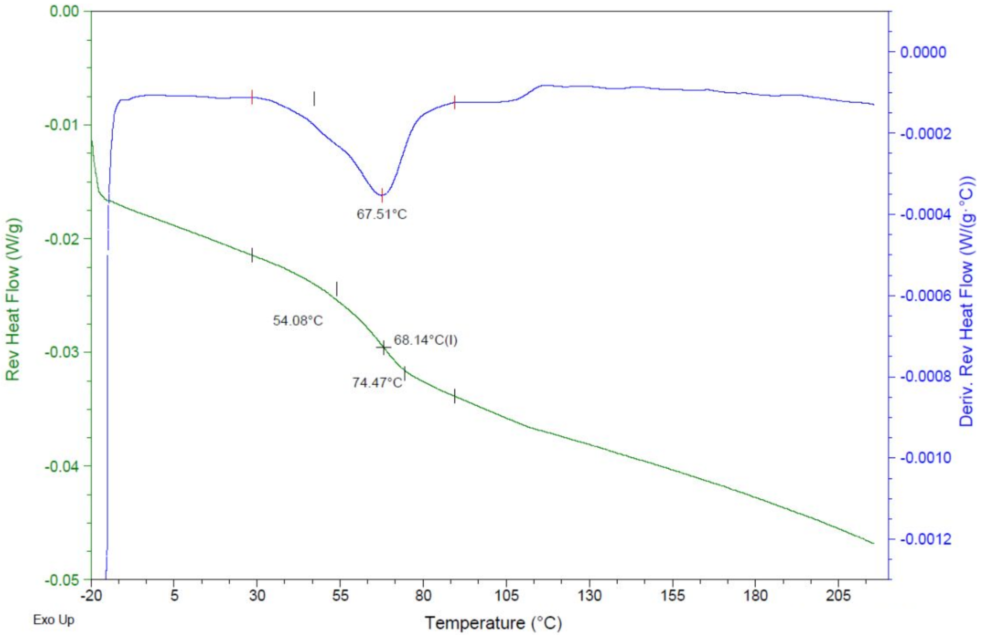

Figure S10. MDSC measurement of ST-1 prior to submersion in water: 15 mg of granulate is loaded into a pan and equilibrated for 5 min at – 20 °C after which it is heated up to 200 °C with a ramp of 2 °C/min with a +/- 1.5 °C modulation every 60 seconds.

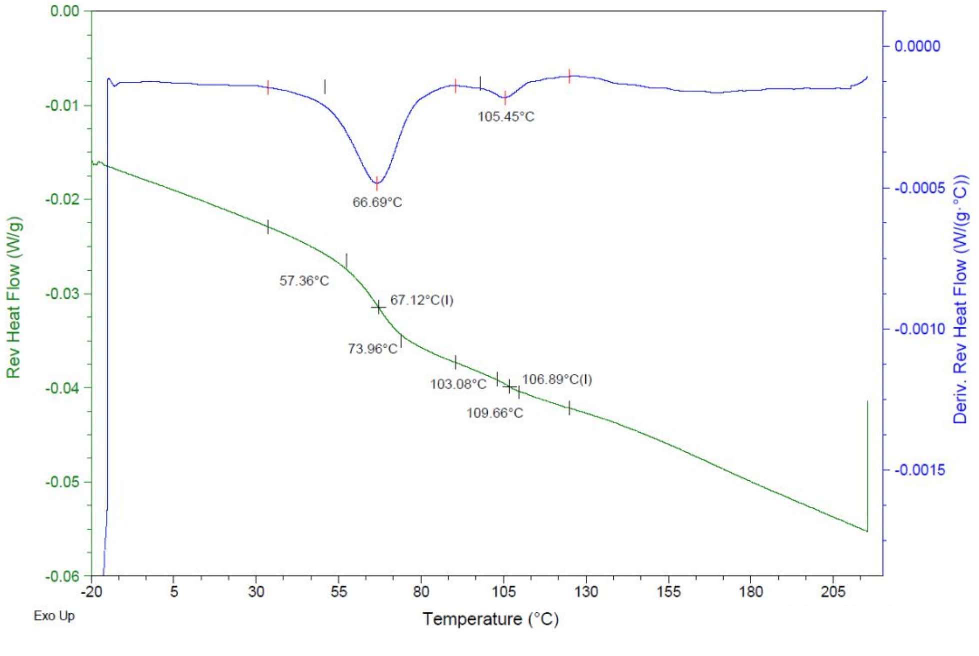

Figure S11. MDSC measurement of ST-1 after 7 days of exposure in 55 °C water: 15 mg of granulate is loaded into a pan and equilibrated for 5 min at – 20 °C after which it is heated up to 200 °C with a ramp of 2 °C/min with a +/- 1.5 °C modulation every 60 seconds.

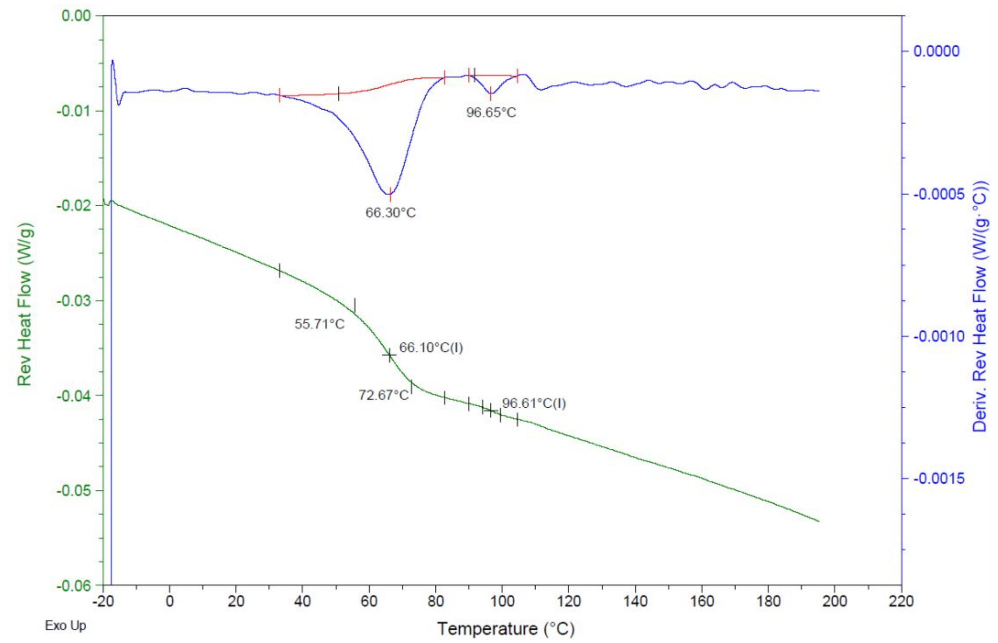

Figure S12. MDSC measurement of ST-1 after 10 days of exposure in 55 °C water: 15 mg of granulate is loaded into a pan and equilibrated for 5 min at – 20 °C after which it is heated up to 200 °C with a ramp of 2 °C/min with a +/- 1.5 °C modulation every 60 seconds.

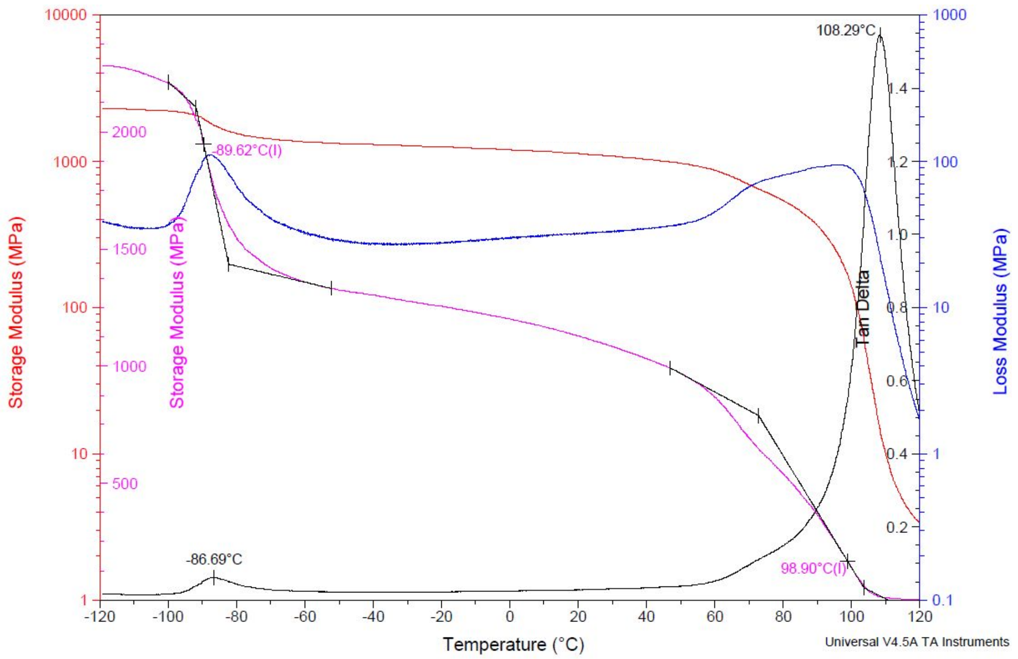

Figure S13. DMA measurement of the LN-2 sample starting at -120 °C for 1 min and heating to 120 °C with a ramp of 2 °C/min after which the sample is kept at 120 °C for 1 min. The measurement is performed with an amplitude of 25 μm and a frequency of 1 Hz.

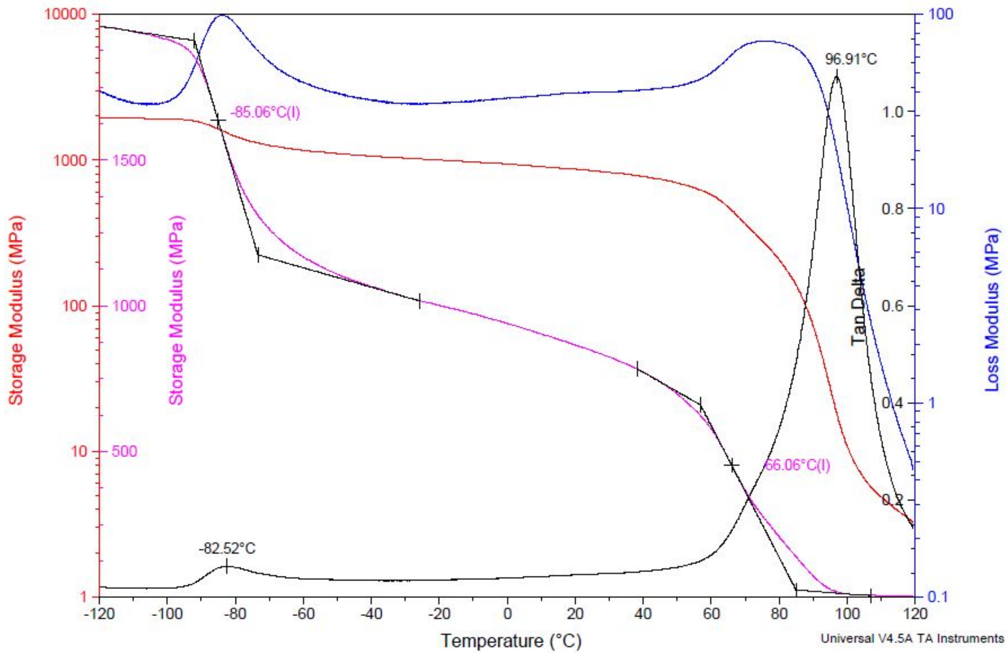

Figure S14. DMA measurement of the LN-3 sample starting at -120 °C for 1 min and heating to 120 °C with a ramp of 2 °C/min after which the sample is kept at 120 °C for 1 min. The measurement is performed with an amplitude of 25 μm and a frequency of 1 Hz.

9. Supplementary TEM images and SAXS measurements

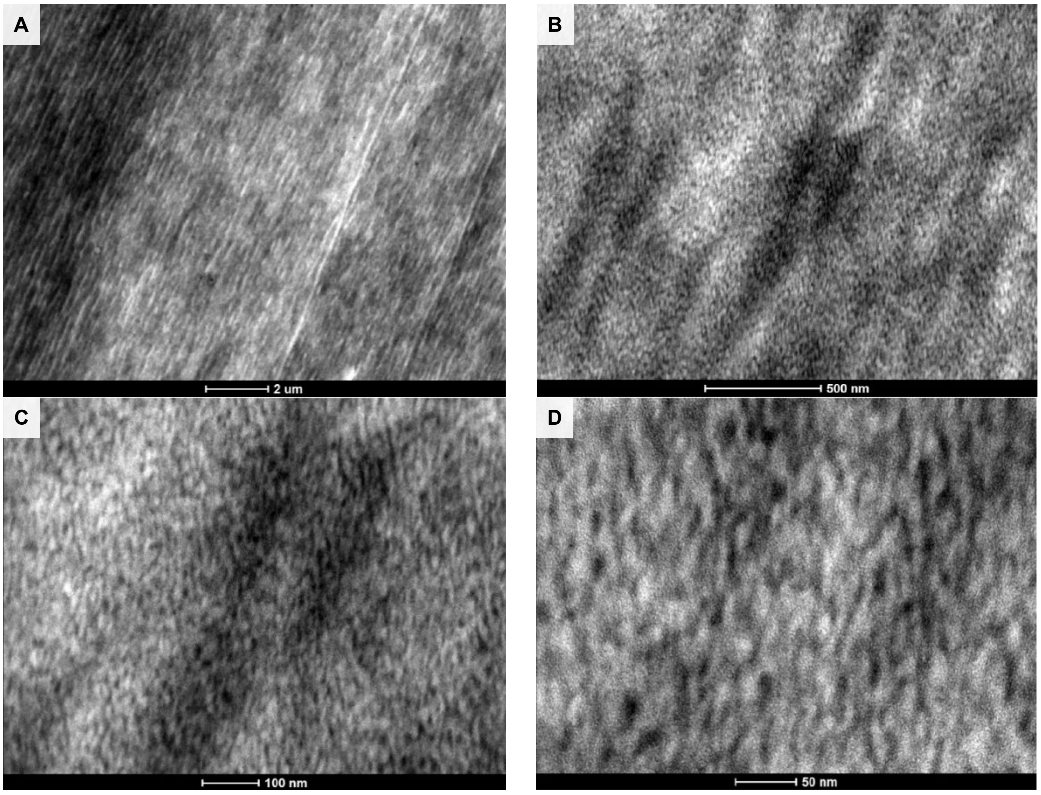

Figure S15. TEM images measured on OsO4 stained microtome cut specimen produced from 2 mm optical plaques for sample LN-2 at different magnifications.

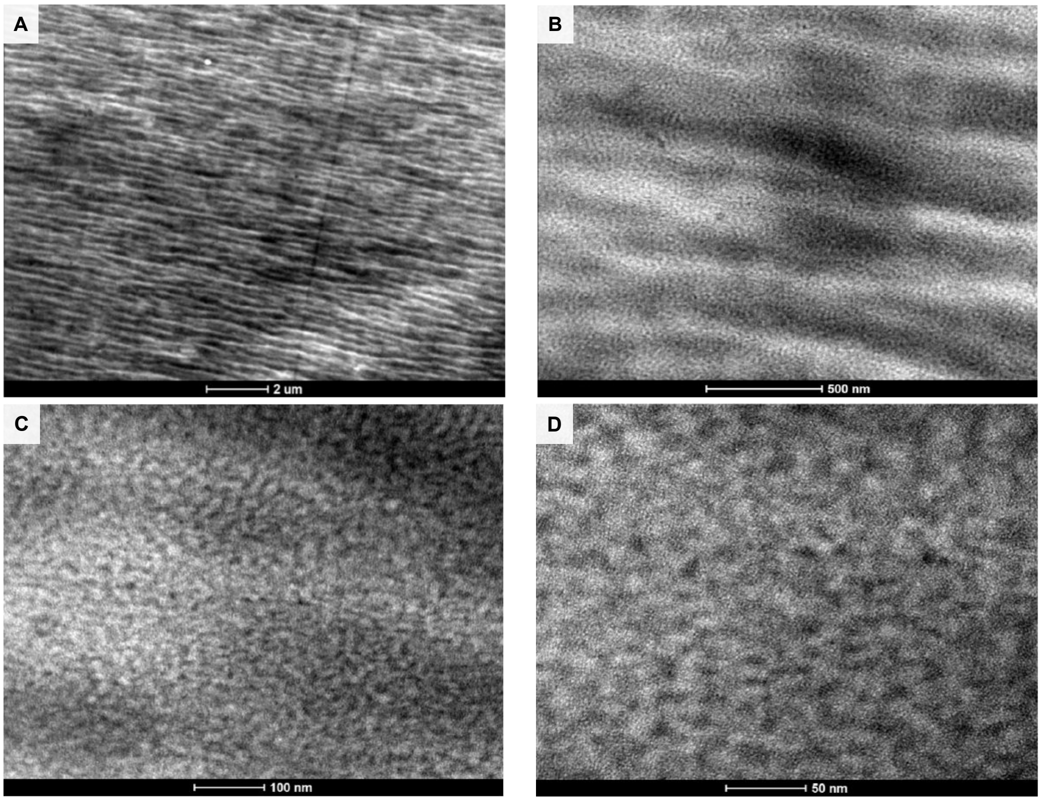

Figure S16. TEM images measured on OsO4 stained microtome cut specimen produced from 2 mm optical plaques for sample LN-3 at different magnifications.

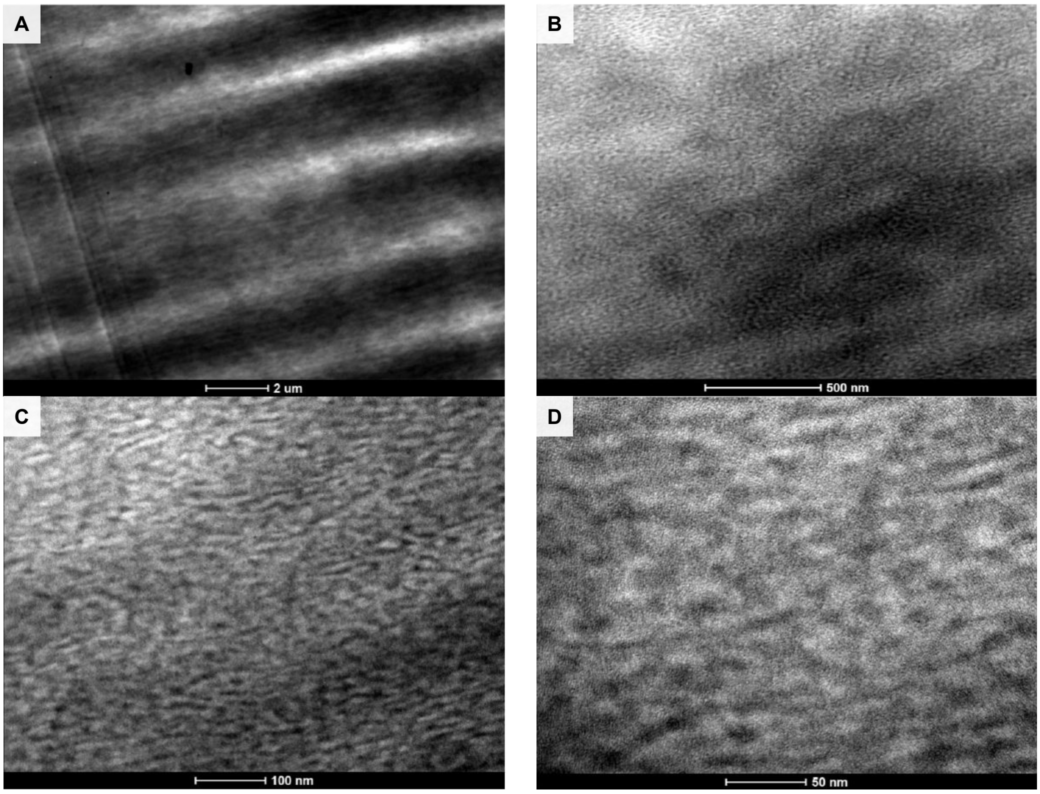

Figure S17. TEM images measured on OsO4 stained microtome cut specimen produced from 2 mm optical plaques for sample LN-5 at different magnifications.

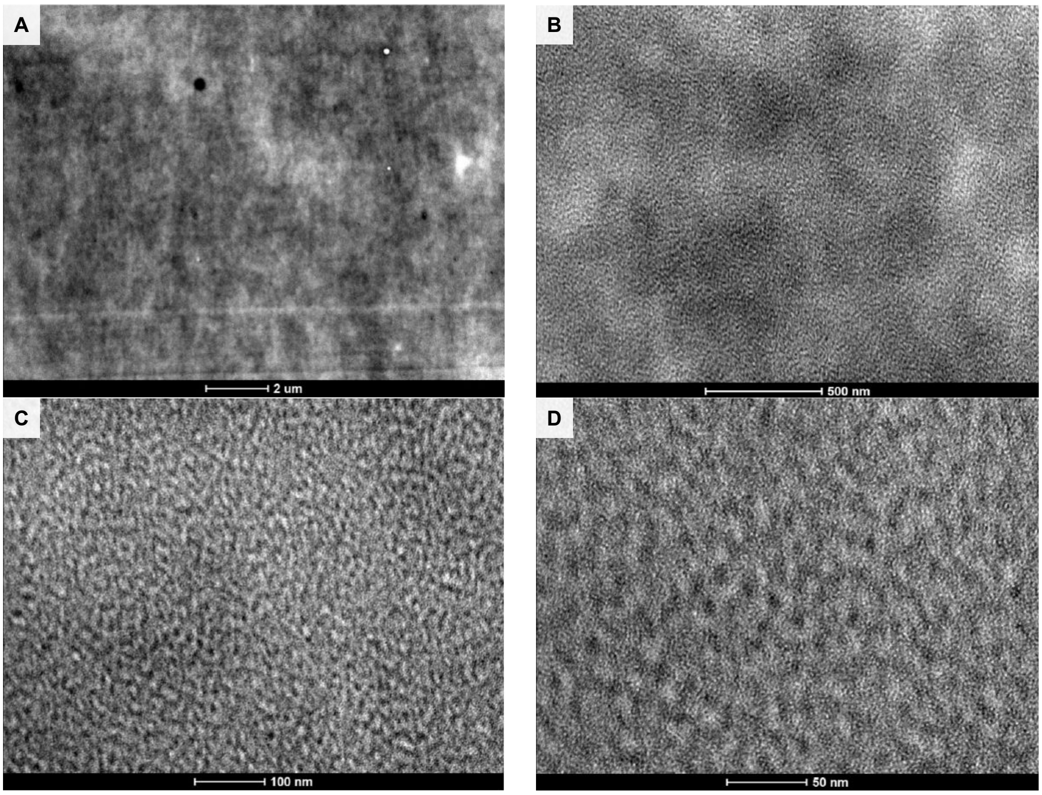

Figure S18. TEM images measured on OsO4 stained microtome cut specimen produced from 2 mm optical plaques for sample LN-7 at different magnifications.

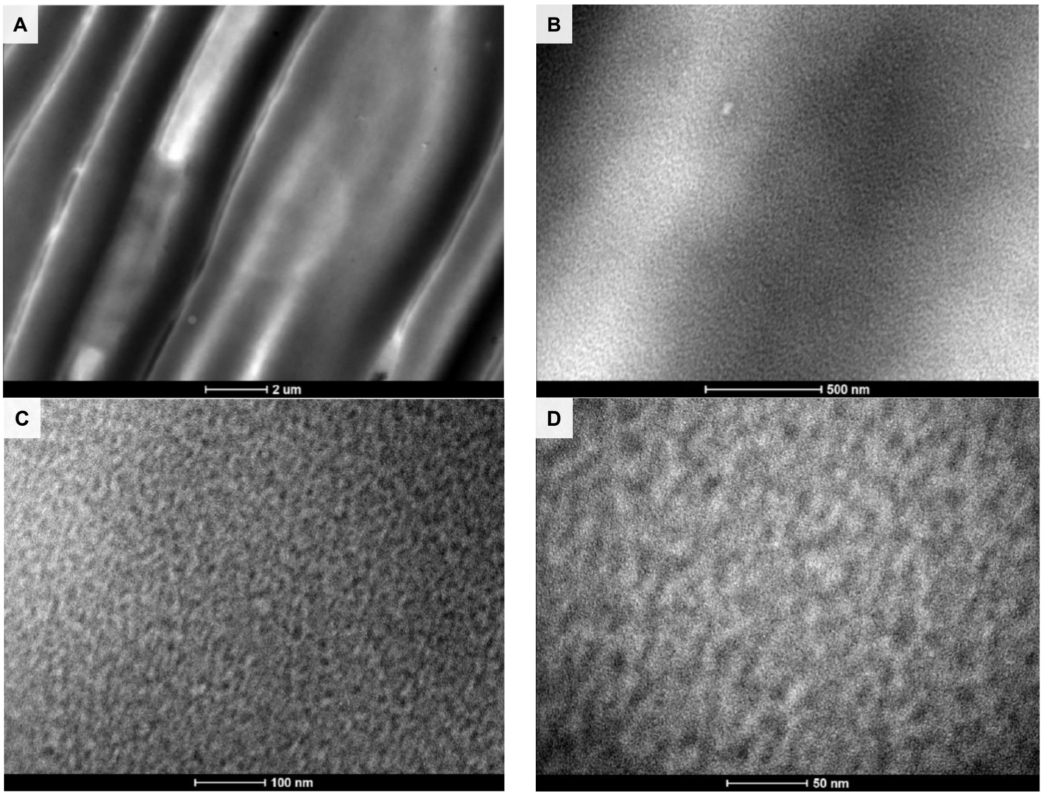

Figure S19. TEM images measured on OsO_4 stained microtome cut specimen produced from 2 mm optical plaques for sample LN-1 at different magnifications.

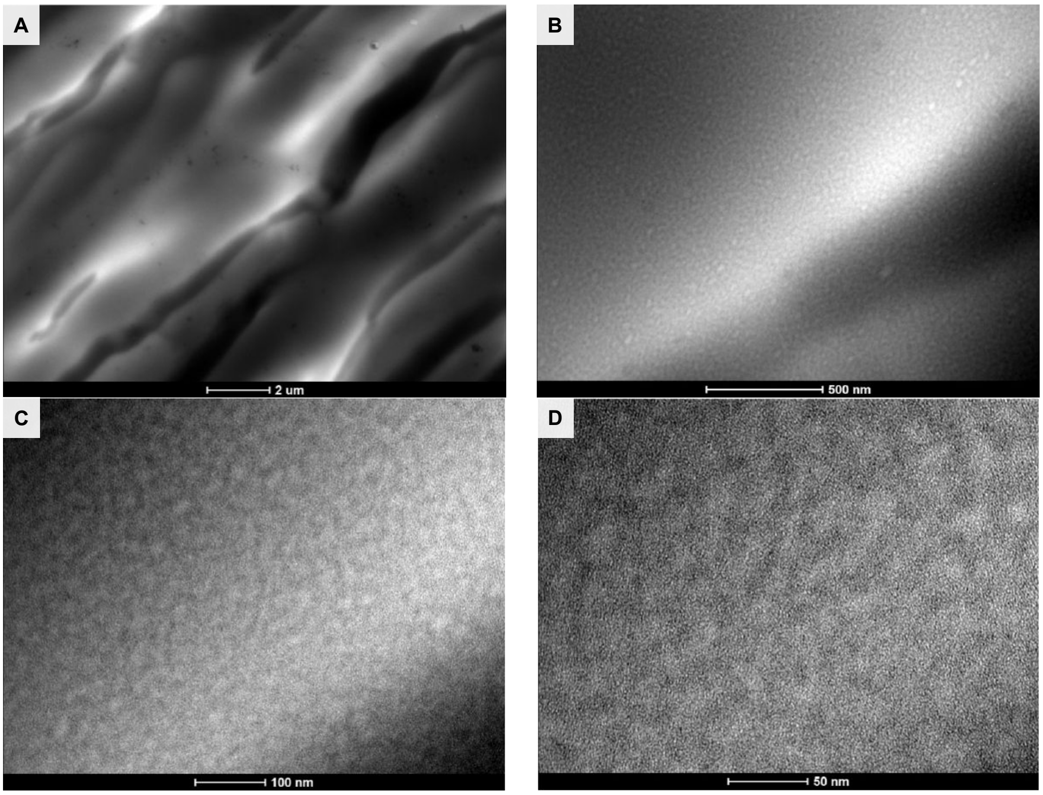

Figure S20. TEM images measured on OsO_4 stained microtome cut specimen produced from 2 mm optical plaques for sample LN-4 at different magnifications.

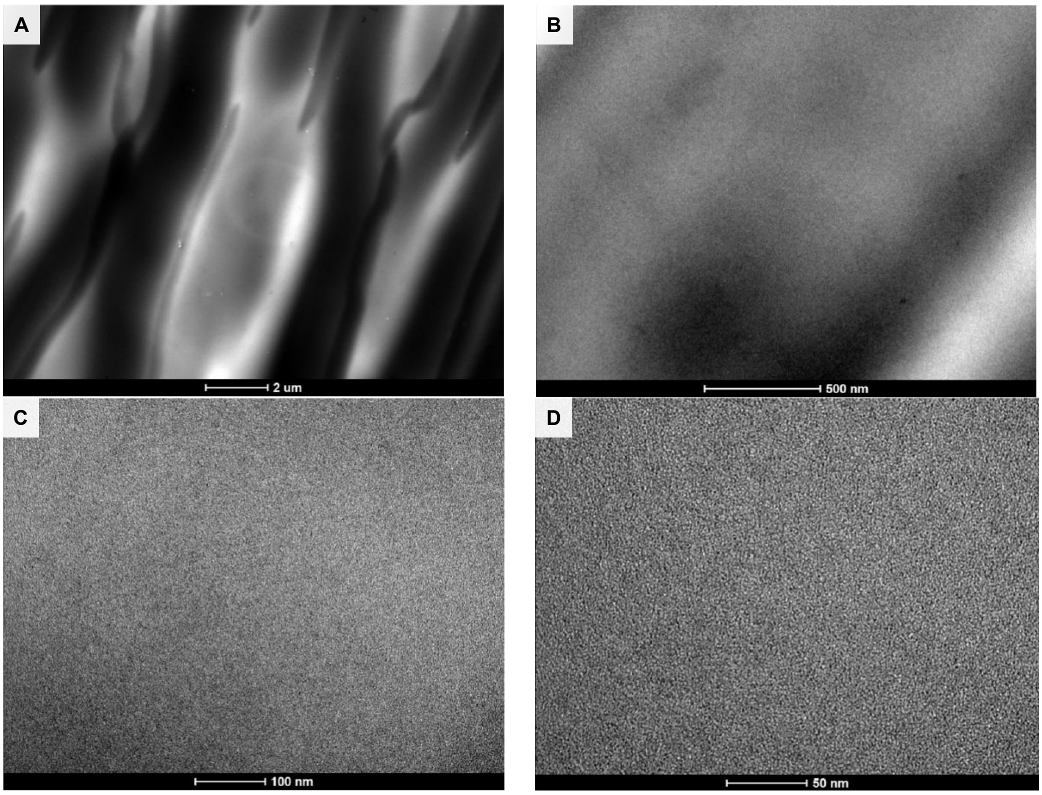

Figure S21. TEM images measured on OsO4 stained microtome cut specimen produced from 2 mm optical plaques for sample LN-6 at different magnifications.

Figure S22 presents SAXS patterns of samples LN-1, LN-2, LN-3, LN-4 and LN-7. In all cases, a primary Bragg reflection is visible, indicating a microphase-separated structure of the block-copolymers. The morphology depends strongly on the sample. LN-2 (Figure S22A) shows a clear primary Bragg at 0.0180 Å^{-1} (q_0), corresponding to a repeat distance of $2\pi/q_0 \approx 34.9$ nm. A very broad secondary reflection at 0.043-0.057 Å^{-1} (q_1), which is consistent with $q_1 = \sqrt{7} \cdot q_0$, expected for a hexagonal structure.[1] Sample LN-3 (Figure S22B) shows Bragg peaks at 0.0259 Å^{-1} (q_0) and at 0.045-0.055 Å^{-1} (q_1), which corresponds to a lamellar structure ($q_1 = 2 \cdot q_0$) with a repeat distance of 24.3 nm. LN-7 (Figure S22C) shows a primary Bragg peak at 0.0275 Å^{-1}, corresponding to a repeat distance of 22.8 nm, but lacks any secondary peaks. Therefore, its microphase-separated structure is a random one without long-range order. LN-1 (Figure S22D) shows Bragg peaks at 0.0227 Å^{-1} (q_0) and at 0.039-0.045 Å^{-1} (q_1). These positions are consistent with both a hexagonal and lamellar morphology and a repeat distance of 27.7 nm. Lastly, LN-4 (Figure S22E) only shows a primary Bragg peak at 0.0216 Å^{-1} (corresponding to a repeat distance of 29.1 nm) without secondary Bragg peaks and, therefore, lacks any morphology with long-range order.

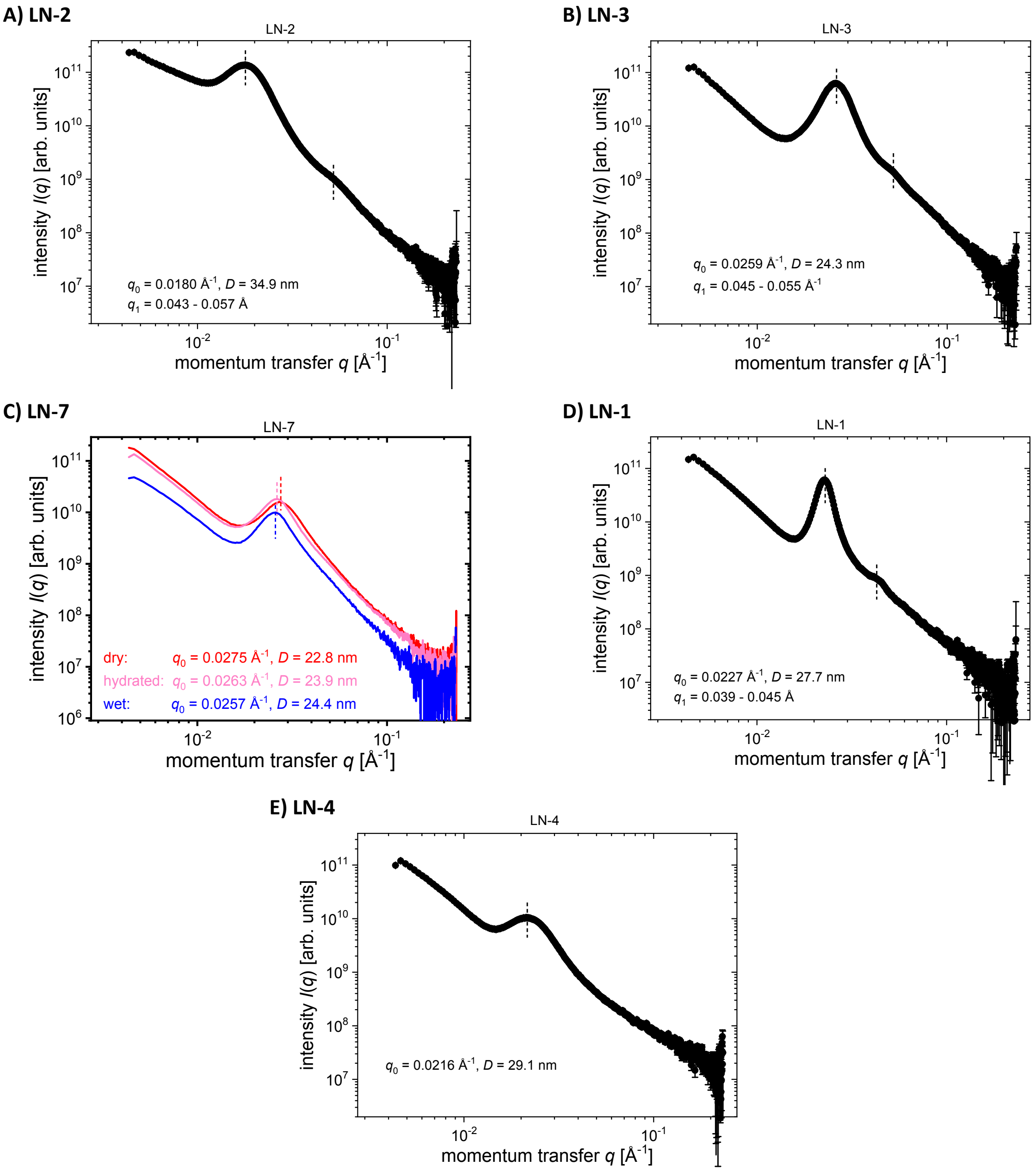

Figure S22. SAXS measurements for the different linear polymer samples.

Sample LN-7 (Figure S22C) was measured under hydrated conditions, too. Exposed to an atmosphere with high relative humidity, the primary Bragg peak shifts slightly to a q-value of 0.0263 Å^{-1}. As compared to the dry structure, the repeat distance of the disordered microphase-separated structure increased slightly to a value of 23.9 nm. Immersed in water, the repeat distance increased further to 24.4 nm.

10. Regression-extrapolation

10.1. First assessment.

In the first assessment, the 7 different functions in Table S3 were used to regress a 336 hours of measurement data for polymer LN-1 and polymer LN-2. The results are shown in Figure S23 and Figure S24. Due to the poor performance of the polynomial in both examples, it was left out of the further analysis. Due to the better fit of the first order exponential function, compared to the second order, only the first order exponential was considered in follow-up analysis. However, an additional parameter was introduced so that the preexponential factor and the constant are not necessarily identical. As the second order rational function gave an identical fit as the shifted first order in case of LN-1, and a slightly better fit in LN-2, this function was selected for the final analysis.

Table S3. Different mathematical descriptions applied in the primary selection of candidate functions for regression and extrapolation of the loss of transparency as a function of time.

Description	Function	Number
Power law	$\Delta Trans = a \cdot t^b$	(S1)
First degree exponential	$\Delta Trans = a(1 - e^{-bt})$	(S2)
Second degree exponential	$\Delta Trans = a(1 - e^{-bt^2+ct+d})$	(S3)
Third order polynomial	$\Delta Trans = at^3 + bt^2 + ct + d$	(S4)
First order rational	$\Delta Trans = a - \frac{b}{t}$	(S5)
Shifted first order rational	$\Delta Trans = a - \frac{b}{t + c}$	(S6)
Second order rational	$\Delta Trans = a - \frac{bt + c}{dt^2 + et + f}$	(S7)

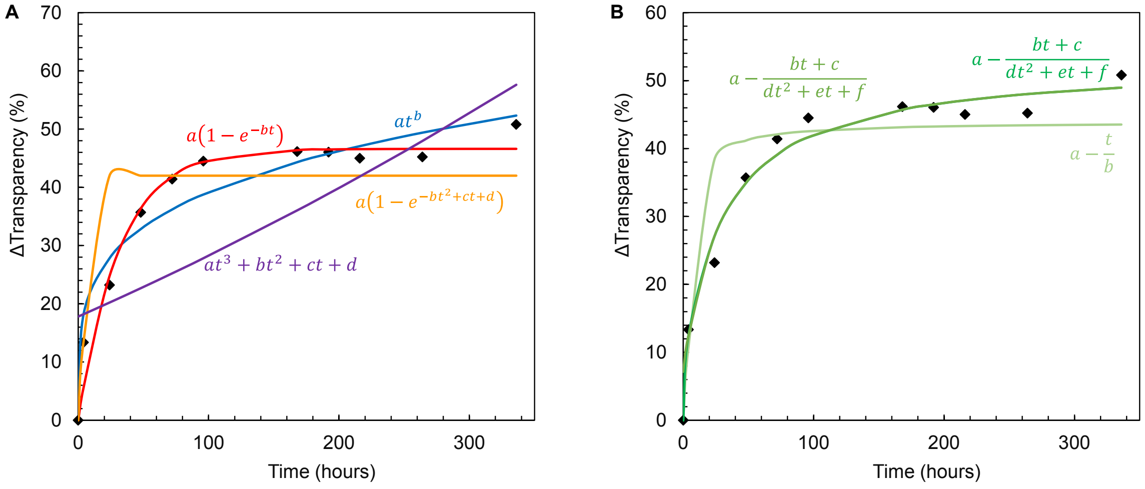

Figure S23. Regression results of the functions in Table S3 applied to 336 hours of measurement data of polymer LN-1: (**A**) regression to exponential, power law and polynomial functions and (**B**) regression to rational functions.

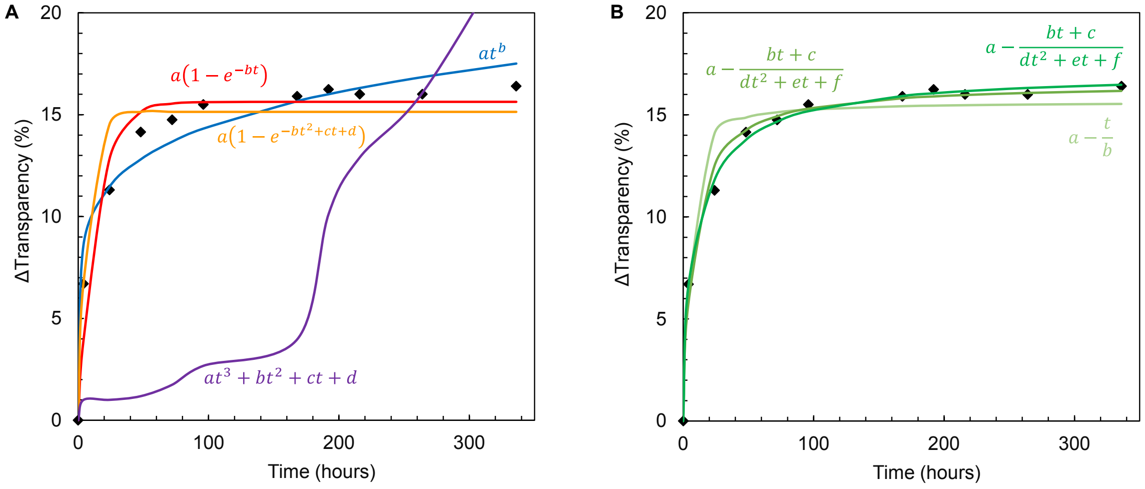

Figure S24. Regression results of the functions in Table S3 applied to 336 hours of measurement data of polymer LN-2: (**A**) regression to exponential, power law and polynomial functions and (**B**) regression to rational functions.

10.2. Demonstrating experiments

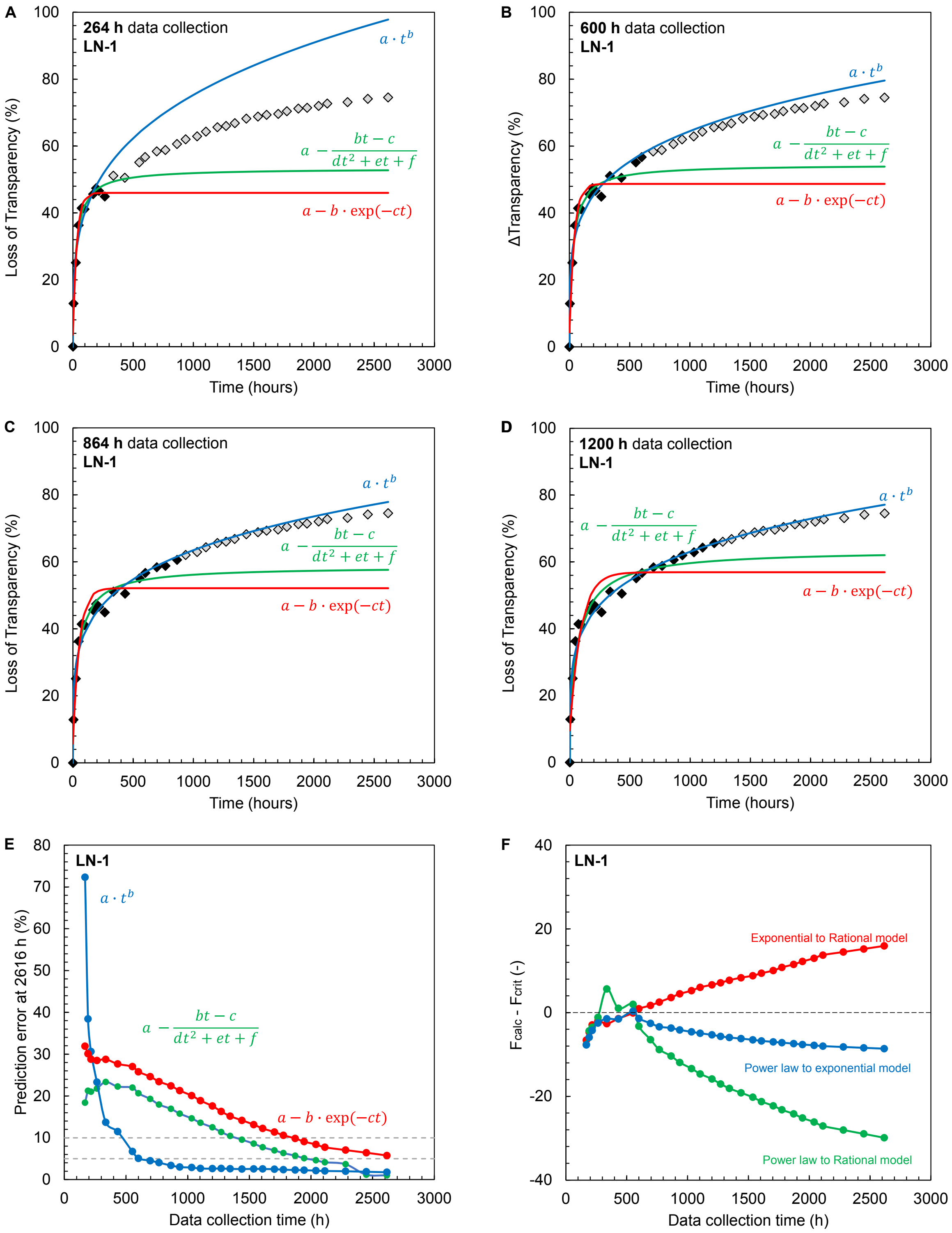

Figure S25. Regression-extrapolation of the loss of transparency in a 2 mm optical plaque of polymer LN-1 immersed in 55 °C salt-free demineralized water based on measurement data spread out over 2616 hours. Regression and extrapolation of the candidate functions after (**A**) 264 hours (**B**) 600 hours (**C**) 864 hours and (**D**) 1200 hours of measurement. (**E**) Absolute prediction error for each of the descriptions as a function of data collection time. (**F**) Statistical F-test as a function of data collection time.

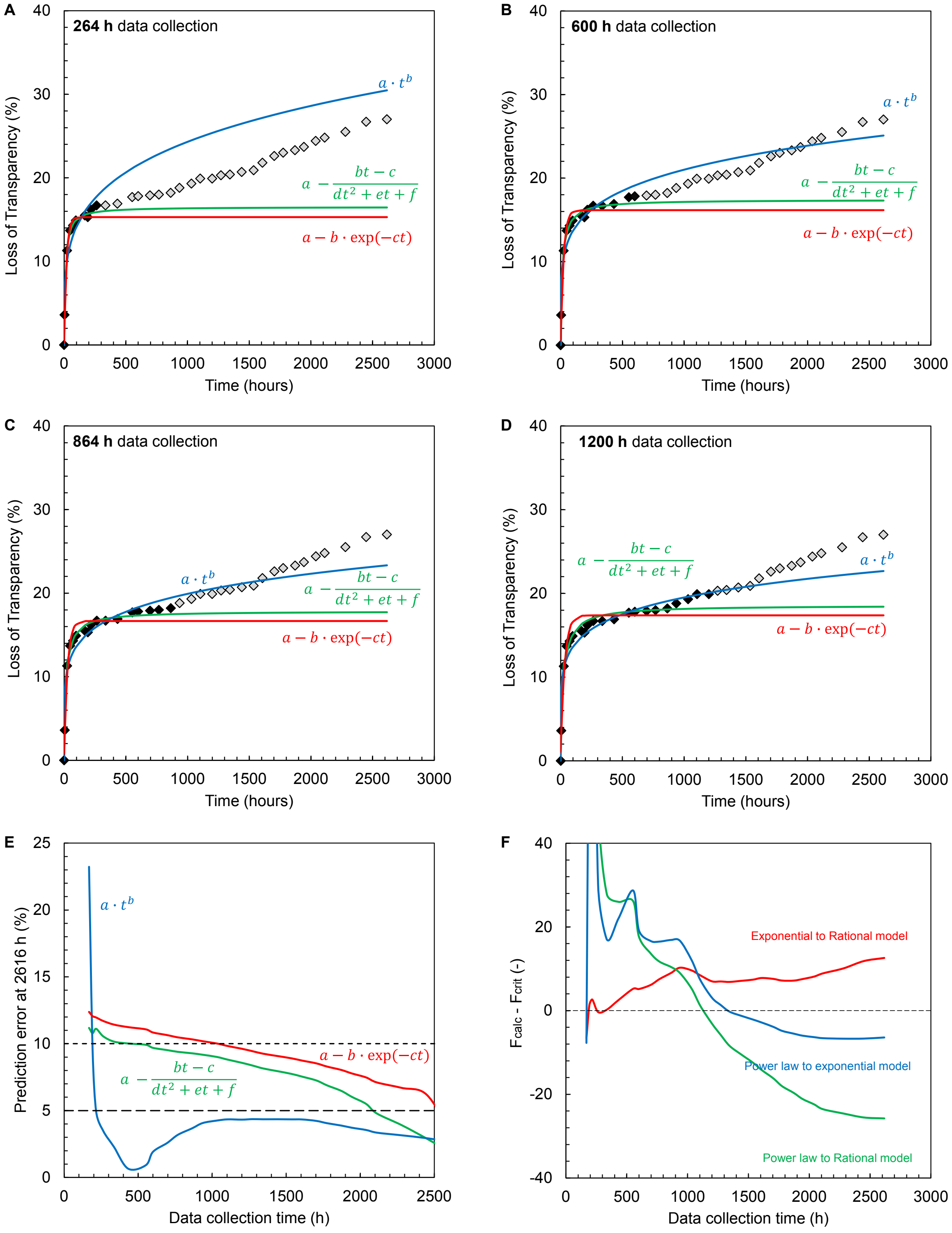

Figure S26. Regression-extrapolation of the loss of transparency in a 2 mm optical plaque of polymer LN-2 immersed in 55 °C salt-free demineralized water based on measurement data spread out over 2616 hours. Regression and extrapolation of the candidate functions after (**A**) 264 hours (**B**) 600 hours (**C**) 864 hours and (**D**) 1200 hours of measurement. (**E**) Absolute prediction error for each of the descriptions as a function of data collection time. (**F**) Statistical F-test as a function of data collection time.

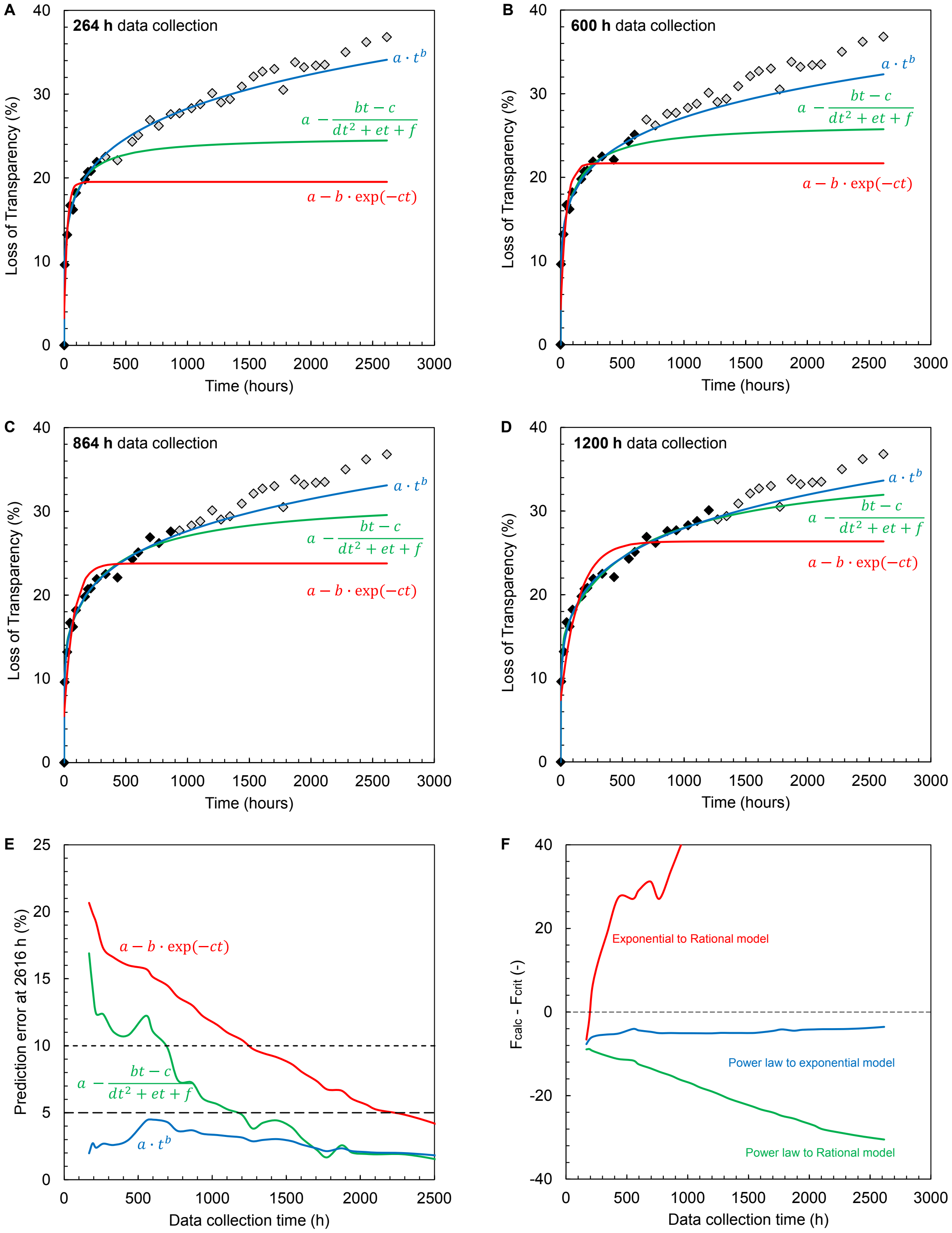

Figure S27. Regression-extrapolation of the loss of transparency in a 2 mm optical plaque of polymer LN-3 immersed in 55 °C salt-free demineralized water based on measurement data spread out over 2616 hours. Regression and extrapolation of the candidate functions after (**A**) 264 hours (**B**) 600 hours (**C**) 864 hours and (**D**) 1200 hours of measurement. (**E**) Absolute prediction error for each of the descriptions as a function of data collection time. (**F**) Statistical F-test as a function of data collection time.

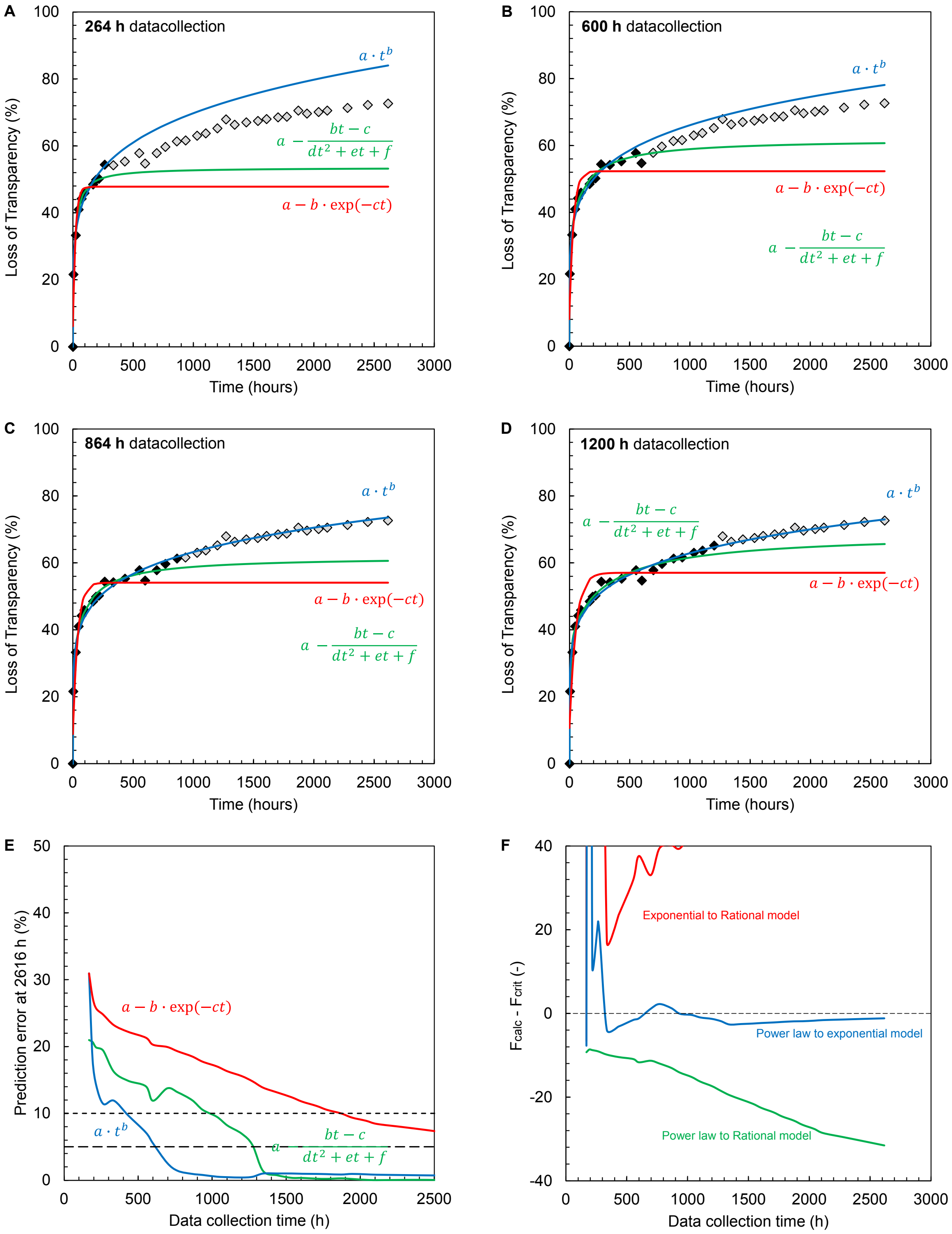

Figure S28. Regression-extrapolation of the loss of transparency in a 2 mm optical plaque of polymer LN-4 immersed in 55 °C salt-free demineralized water based on measurement data spread out over 2616 hours. Regression and extrapolation of the candidate functions after (**A**) 264 hours (**B**) 600 hours (**C**) 864 hours and (**D**) 1200 hours of measurement. (**E**) Absolute prediction error for each of the descriptions as a function of data collection time. (**F**) Statistical F-test as a function of data collection time.

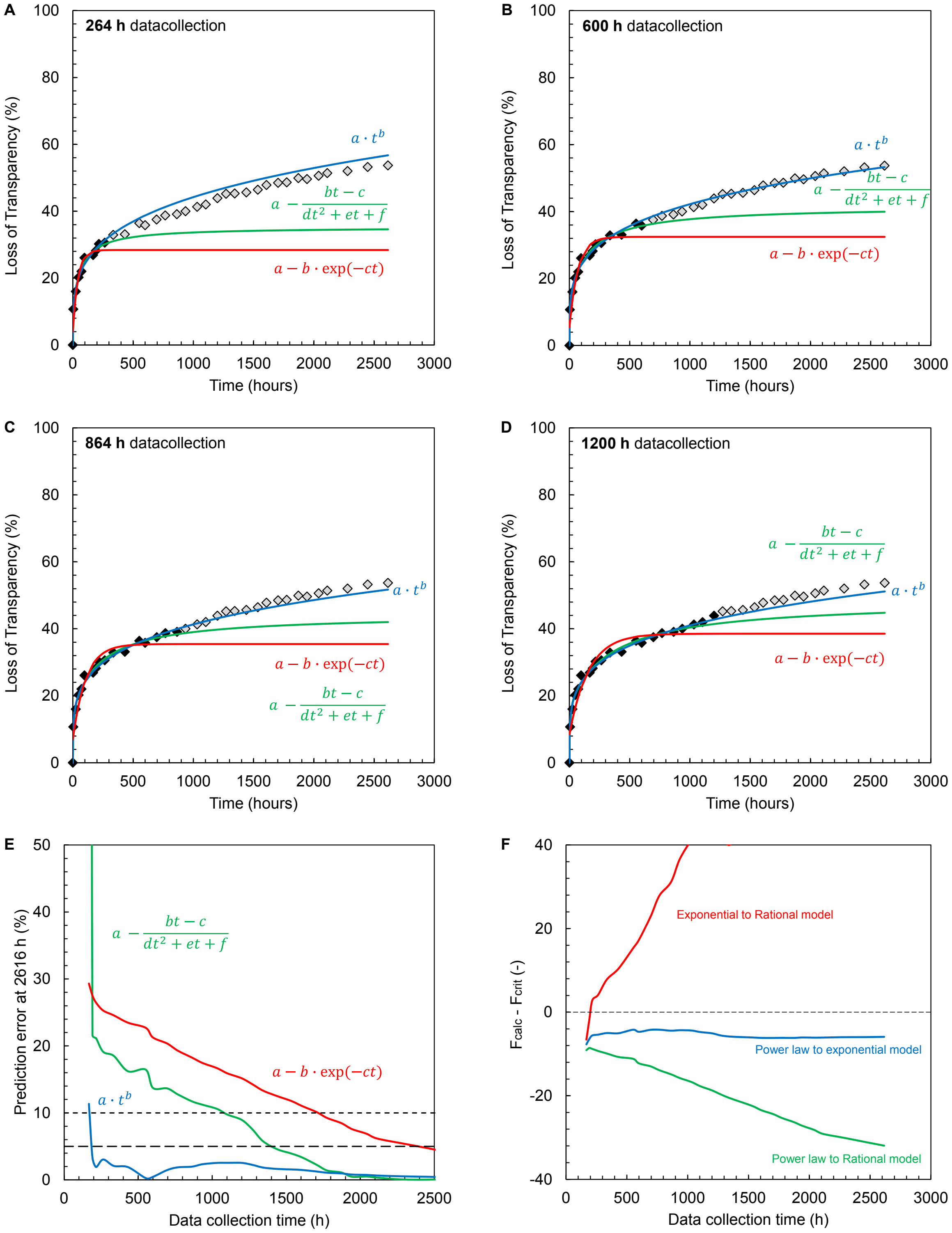

Figure S29. Regression-extrapolation of the loss of transparency in a 2 mm optical plaque of polymer LN-5 immersed in 55 °C salt-free demineralized water based on measurement data spread out over 2616 hours. Regression and extrapolation of the candidate functions after (**A**) 264 hours (**B**) 600 hours (**C**) 864 hours and (**D**) 1200 hours of measurement. (**E**) Absolute prediction error for each of the descriptions as a function of data collection time. (**F**) Statistical F-test as a function of data collection time.

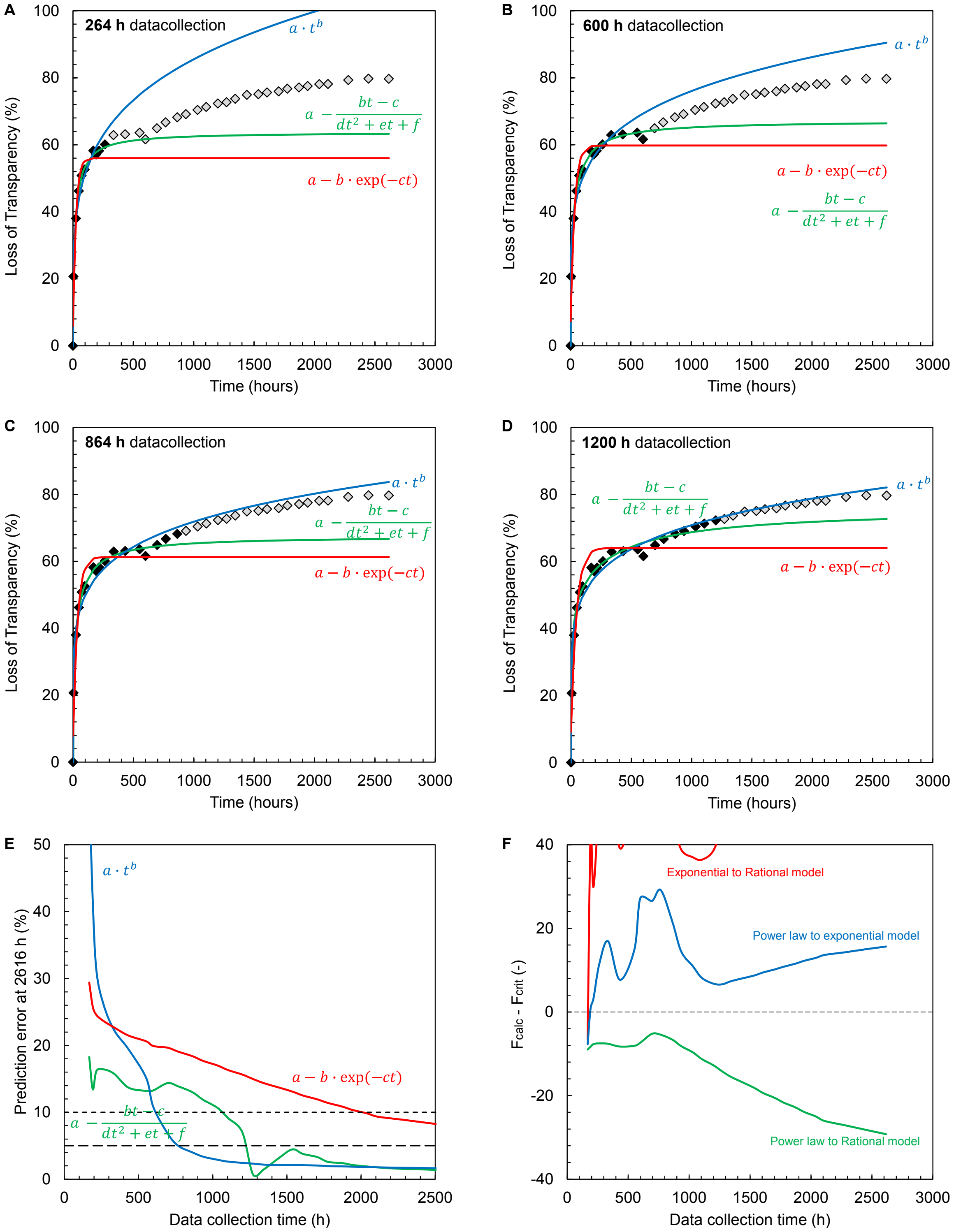

Figure S30. Regression-extrapolation of the loss of transparency in a 2 mm optical plaque of polymer LN-6 immersed in 55 °C salt-free demineralized water based on measurement data spread out over 2616 hours. Regression and extrapolation of the candidate functions after (**A**) 264 hours (**B**) 600 hours (**C**) 864 hours and (**D**) 1200 hours of measurement. (**E**) Absolute prediction error for each of the descriptions as a function of data collection time. (**F**) Statistical F-test as a function of data collection time.

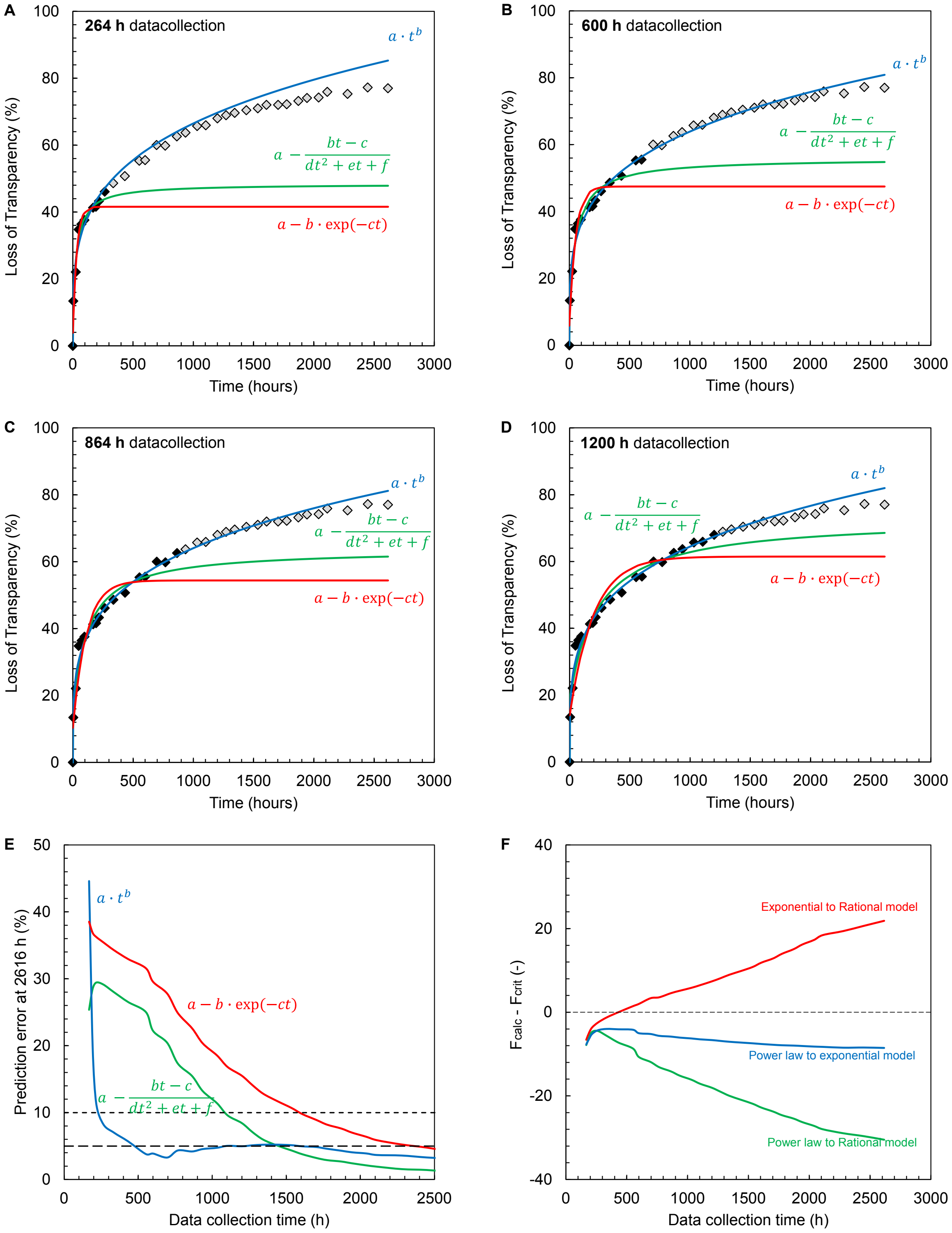

Figure S31. Regression-extrapolation of the loss of transparency in a 2 mm optical plaque of polymer LN-7 immersed in 55 °C salt-free demineralized water based on measurement data spread out over 2616 hours. Regression and extrapolation of the candidate functions after (**A**) 264 hours (**B**) 600 hours (**C**) 864 hours and (**D**) 1200 hours of measurement. (**E**) Absolute prediction error for each of the descriptions as a function of data collection time. (**F**) Statistical F-test as a function of data collection time.

11. References

(1) Hamley, I. W.; Castelletto, V., Small-angle scattering of block copolymers: in the melt, solution and crystal states. *Progress in Polymer Science* **2004,** *29* (9), 909-948, DOI: 10.1016/j.progpolymsci.2004.06.001.
